# Breaking the paradigms of residual categories and neglectable importance of non-used resources: the “vital” traditional knowledge of non-edible mushrooms and their substantive cultural significance

**DOI:** 10.1186/s13002-021-00450-3

**Published:** 2021-04-21

**Authors:** Amaranta Ramírez-Terrazo, E. Adriana Montoya, Roberto Garibay-Orijel, Javier Caballero-Nieto, Alejandro Kong-Luz, Claudia Méndez-Espinoza

**Affiliations:** 1 Posgrado en Ciencias Biológicas, Universidad Nacional Autónoma de México, Apartado Postal 70-153, C.P. 04510 CDMX, México; 2 Instituto de Biología, Universidad Nacional Autónoma de México, Apartado Postal 70-153, C.P. 04510 CDMX, México; 3 Centro de Investigación en Ciencias Biológicas, Universidad Autónoma de Tlaxcala, Km 10.5 Autopista San Martín Texmelucan-Tlaxcala, Ixtacuixtla Tlaxcala, CP. 90120 México; 4 Centro Nacional de Investigación Disciplinaria en Conservación y Mejoramiento de Ecosistemas Forestales, Instituto Nacional de Investigaciones Forestales Agrícolas y Pecuarias, Av. Progreso 5, Barrio de Santa Catarina, Coyoacán, CP 04010 CDMX, México

**Keywords:** Non-edible mushrooms, Local knowledge, Cultural importance, Ethnomycology, Toxic mushrooms, Local classification, Non-used resources

## Abstract

**Introduction:**

One of the main goals of ethnomycological studies has been understanding the role of wild edible mushrooms (WEM) in diverse cultures. To accomplish such a purpose, the local knowledge of WEM and their cultural importance have been evaluated and compared using qualitative and quantitative methods. However, few studies have documented these aspects in non-edible mushrooms, because they are considered to be in a category of residual cultural importance. To make up for this lack of investigation, this paper analyzes the traditional knowledge of non-edible mushrooms to understand their cultural role and break it down to its components. The analysis of this topic shows how this knowledge represents a good strategy to prevent mushroom intoxications in humans.

**Methods:**

This study was carried out in two communities residing in La Malintzi National Park, Tlaxcala, Mexico. Mushroom species indicated as non-edible were collected during 13 ethnomycological expeditions and seven requests. To get an insight into the local knowledge about these mushrooms, we used ethnographic techniques, 91 free listings and 81 semi-structured interviews.

**Results:**

In total, we collected 178 specimens of wild mushrooms recognized as non-edible by locals, which corresponded to 103 species belonging to 45 genera. People who participated in the study had a vast and deep understanding of non-edible mushrooms. For them, the most important species were *Amanita muscaria*, *Neoboletus* aff. e*rythropus*, *Xerocomellus chrysenteron*, and *Suillus tomentosus.* Two uses were the most mentioned by respondents: as an insecticide and for medicinal purposes. Of note, however, is that *A. muscaria* was reported as edible years ago. To avoid possible intoxication, all non-edible mushrooms were included in the general category of “poisonous mushrooms.” Non-edible species are seen as a cosmogonic counterpart (“twins”) of the edible species that they resemble. We obtained 101 specific recognition criteria, useful only when comparing paired species: edible vs non-edible. The most culturally important non-edible groups were differentiated by clear and precise characteristics, which were reflected in the nomenclature and allowed their classification into specific ethnotaxa.

**Conclusions:**

We found that non-used resources can be the object of a deep traditional knowledge and have a vast cultural importance. In the case of wild non-edible mushrooms in particular: the species are named; they are the subject of vast traditional knowledge which is based on their edible/non-edible duality; this knowledge is widespread but has limited consensus, there is little lexical retention; and this knowledge is vital to avoid fatal intoxications. In consequence, both deadly species and species that share similarities with the most important edible mushrooms have a high cultural importance.

**Supplementary Information:**

The online version contains supplementary material available at 10.1186/s13002-021-00450-3.

## Background

In its early stages, ethnobiology had a utilitarian approach, with a special interest in natural resources that have potential medicinal or dietary uses. Throughout time, this approach has been predominant [1]. Even though living organisms have a meaning beyond the realm of utility [2–4] in terms of local knowledge and cultural importance, species that serve no purpose and without local names are commonly ignored in ethnobiological studies [2]. Human cultures build their lore upon people’s perception and interpretation of the elements of their environment, supported by their beliefs (*kosmos*), knowledge (*corpus*), and practices (*praxis*) [5].

There is a universal trend within cultures to organize and classify phenomena that can be perceived through lived experiences; therefore, structural guidelines based on comparisons can lead to the identification of groups of organisms [6]. People also recognize the organisms according to common traits. In that sense, morphological similarities and dissimilarities are the basis of ethnobiological classifications, leading to hierarchical categories that are orally transmitted as local names [7], and in this sense, the aspect of utility is of secondary relevance [8]. Other ideas suggest, however, that knowledge production requires an energy investment that will make that knowledge useful, adaptable, and of interest for a given culture [9].

An ethnobiological paradigm has emerged from these points of view: “Useless organisms do not have names and are not classified.” This leads to “empty taxonomic spaces,” where all non-used species are regrouped under the name “residual categories or residual taxa.” These categories encompass among others (1) species that are related to other useful species, (2) rare species, and (3) species with only marginal cultural relevance [8–13].

Modern ethnobiological research indicates that local classification systems are multidimensional, where cognitive, psychological, symbolic, utilitarian, and even cosmological aspects interact [3, 12]. Within classification systems, there are diverse grouping strategies such as lateral, hierarchical, and functional linkages. Altogether, the structure and meaning of the names are fundamental to analyzing local classifications [3].

Some ethnomycological studies, including those of non-edible mushroom species or those having negligible nutritional value have shown how people from different areas in the world, consider them culturally worthless [11, 13–20]. Edibility is not an attribute that is inherent to a given species; however, it is determined by the culinary practices of every culture, by the processing and conservation methods, by the forms and amounts of ingestion, and by symbolic associations [13, 15, 17, 18].

Wild mushroom use is of great nutritional [14, 21], economic [22, 23], social [24], and cultural importance [25]. Nevertheless, for non-expert users, consuming wild mushrooms is potentially very risky—possibly even leading to death [17, 26, 27]. Knowledge of these natural resources is mainly possessed and developed by the communities who live in the vicinity of wild mushrooms, but these areas have changed so much in recent years that certain elements that were key to the recognition and identification of toxic and lethal species have been lost, resulting in significant public health risks [16].

The most important classification for wild mushrooms is as edible or non-edible, with the latter considered a residual category [11, 13] in which only species that are closely related to notable edible species have names [28]. In many cultures, wild non-edible mushrooms (WNEM) are collectively identified under terms like *bol lu’* (stupid/crazy mushroom) (tseltal, Chiapas, Mexico) [13, 29], *pitzunanácatl* (rabies mushrooms) (nahuatl, Tlaxcala, Central Mexico) [20, 26], *lu´* (vagina) (tseltal, Chiapas Mexico) [10]), *ã*^*h*^*kilo* (mushroom) (hotï, Venezuela) [30], *gauku ma dorou* (bad gauku) (Nuaulu, Indonesian Islam of Seram) [11], *uccanabe* (non-edible mushroom) (Solega, Biligiri, India) [2], *awo’oh Satan* and *kiwoh fiyin* (non-edible mushroom) (Belo and Oku respectively, languages from Kilum-Ijim, Cameroon) [31], or *itaikarieya* (essence, spirit or ghost) (Wixaritari, Jalisco, México) [19].

So far, ethnomycological research has focused on assessing the cultural significance of edible mushrooms [32], with frequency and order of mention being the main criteria used to denotate the importance of a mushroom species [33, 34]. Garibay-Orijel et al. [35] proposed a Cultural Significance Index for WEM that entails indicators ad hoc to their nature. This index has proven useful to illustrate the cultural significance of edible mushrooms [36–39]. On the other hand, studies about the importance of toxic mushrooms are scarce [13, 40]. They are nonetheless necessary, especially for mycophilic cultures that are exposed to intoxication risks [17, 41].

Such gaps in knowledge impede the appreciation of the cultural relevance of non-edible and non-used species, some of which are lethal. Therefore, our main objectives for this study were (a) to highlight the importance of traditional knowledge of non-edible mushrooms; (b) to highlight the nature and structure of that knowledge; and (c) to demonstrate the cultural significance of non-edible mushrooms.

## Methods

We used inductive and comparative methods to identify patterns of traditional knowledge of non-edible mushrooms. Our study was carried out in two communities in central Mexico who share the same biological patrimony. Although both have indigenous origins, however, one is now Mestizo (Francisco Javier Mina) and the other is Nahua (San Isidro Buensuceso).

This study analyzed non-edible mushrooms. For this purpose, we defined them as the set of mushrooms that are not part of the local diet. Ethnographic, ethnobiological, and ethnomycological tools focused on non-edible mushrooms, and data analysis was completed by qualitative and quantitative approaches.

### Study sites

**Fig. 1:**
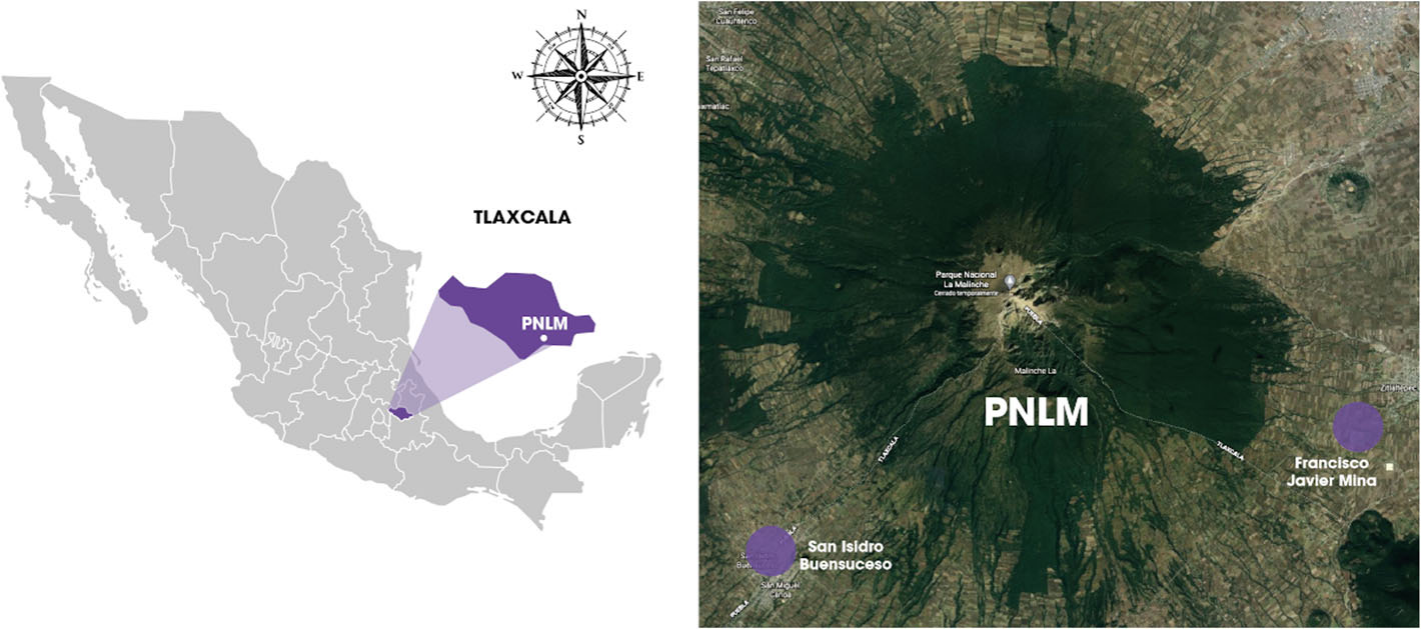
Study area. Purple dots indicate the communities in La Malintzi National Park who participated in the study and from which local knowledge concerning non-edible mushrooms was characterized.

The communities of Francisco Javier Mina and San Isidro Buensuceso, in central Mexico, were selected as study models since their inhabitants have a broad ethnomycological knowledge [20, 26, 32, 34, 42, 43]. Both villages are located in *La Malintzi* Volcano National Park (PNLM), Tlaxcala, Mexico (Fig. 1). Forests with oak, pine, and fir trees are characteristic in the park [44]. There, 91 edible and 16 toxic mushroom species have been recorded, three of them lethal: *Amanita bisporigera* G.F. Atk., *A. virosa* (Fr.) Bertill., and *Galerina marginata* (Batsch) Kühner [45].

Francisco Javier Mina (FJM) (19° 11′ 30″ N, 97° 55′ 45″ W) has an altitude of 2634 m; it belongs to the municipality of Zitlaltépec de Trinidad Sánchez Santos and is located on the southeastern slope of the volcano. Founded at the beginning of the 20th century, it has 1114 people, 0.27% of which are indigenous and 0.45% of which are speakers of an indigenous language [46]. This indicates the loss of indigenous identity. They are manual laborers, housekeepers, traders, and masons, working mostly in Puebla, a neighbor state. During the rainy season, they collect chokecherries and wild mushrooms, which are sold by intermediaries in the central markets of the main nearest cities [47].

San Isidro Buensuceso (SIBS) (19° 09′ 00″ N, 98° 06′ 00″ W) has an altitude of 2619 m, belongs to the municipality of San Pablo del Monte, and is located on the southwestern slope of the volcano. Founded at the end of 19th century [48], the village is home to 8769 people, 73% of which are bilingual (Nahuatl-Spanish). The rest speak only Nahuatl. They have kept many of the characteristic elements of this indigenous group, despite cultural transformations [49]. Currently, a considerable part or the population travels every day to the municipal seats of Puebla and Tlaxcala to work as masons, manual laborers, bus drivers, porters, and seamstresses. Such activities have contributed to transforming the social and cultural patterns, with the result that few people depend on agricultural and forest resources. Nevertheless, during the rainy season, the foraging for mushrooms, *quelites* (wild plants from agricultural crops), and other wild plants is an important endeavor among poor families [26]. Consequently, we hypothesized that, as both populations know, use, and trade edible mushrooms, then they must have a pretty sound knowledge of the species that are either not consumed, poisonous, or a health hazard.

### Data collection and analysis

To get the permission of local civil and traditional authorities for our investigations, we organized meetings to inform them of the nature of the study and our intention to publish the data and images compiled during the research. We also obtained consent from every individual interviewed. Our study was developed according to the code of ethics for research set by the Latin American Society of Ethnobiology [50]. In the rainy seasons (May–October) of 2011 and 2012, we made 14 visits, six to FJM and eight to SIBS. Every visit lasted from 5 to 7 days. For each visit, we were accompanied by expert mushroom foragers, known as *hongueros* (four in FJM and seven in SIBS) [51, 52].

We collected non-edible mushrooms during ethnomycological fieldwork with the help of villagers (six in FJM and seven in SIBS). We made two types of excursions: (1) accompanied by *hongueros*, documenting their procedures for identifying non-edible mushrooms; (2) accompanied by non-specialist villagers. In total, we traveled on 20 trails corresponding to the sites frequented by the local people. We observed and recorded the specimens that are usually not collected, neither by *hongueros* nor by non-specialist villagers, and documented their reactions and attitudes towards such organisms. Then we picked the mushrooms and asked about their specific traits, such as their identification criteria and the name of the edible mushroom to which they seem to bear a resemblance.

Vouchers were characterized according to Cifuentes et al. [53] and Lodge et al. [54]. Taxonomic identification was based on the analysis of micro- and macroscopic traits, using taxonomic keys corresponding to particular genus [55–62]. Authors and nomenclature of the species were consulted in Index Fungorum and MycoBank. Specimens were deposited at the herbarium TLXM.

To evaluate the traditional knowledge about non-edible mushrooms, we used ethnographic approach techniques [63], as well as direct and participant observation [64, 65]. Field note records were prioritized and coded [66]. We also carried out informal interviews, selecting at random 18 interviewees (five in FJM and eight in SIBS), which allowed us to identify the cultural domains used to construct the semi-structured interviews [67–69].

In our second stage, we conducted 81 semi-structured interviews, randomly selecting the interviewees (25 in FJM and 56 in SIBS). The minimum and maximum ages for our interviewees were 7 and 82 years. Interviews addressed the following topics, among others: identification criteria, knowledge transmission, uses, types and symptoms of mycetisms, abundance, traditional remedies for mycetisms, perception and attitudes towards mushrooms and neurotropic mushrooms. The data obtained was organized into categories (one for each similar answer to the interview questions), which we further compared in internal and external pairs [70]. Also, we developed a database and calculated the mention percentage for each category.

To assess the cultural importance of non-edible mushrooms (NEMCI), we considered only those criteria mentioned by more than 10% of our interviewees [26, 34]. The NEMCI indicators were (a) declared cultural importance, (b) mention frequency, and (c) rank ordinal value [26, 34, 35]. Data analysis was performed by means of six basic data matrices [71], from which we estimated frequencies and orders for the next indicators. Mention frequency (MF) was evaluated as a binary qualitative trait: presence (1) and absence (0). For mention order (MO), we used quantitative discrete data (1, 2, 3, 4, 5, … *n*) [72], with which we calculated the rank ordinal value (ROV):

$$ {\mathrm{OVR}}_{\mathrm{spi}}=\sum \limits_{i=1}^n\raisebox{1ex}{$1$}\!\left/ \!\raisebox{-1ex}{$p$}\right. $$

where *p* is the place in the order of the participant’s free listing, *i* is the ethnotaxa spi, and *n* is the number of people that mentioned spi [26, 37]. For the species present in both communities, we estimated the mean mention order (MMO):

$$ \mathrm{MMO}={\sum}_{i=1}^n\mathrm{Sts}/N $$

where Sts indicates the status of the species in the free listing, and *N* is the total number of interviewees [34].

## Results

### Non-edible mushrooms traditional knowledge

#### Non-edible mushrooms identified

In total, we collected 178 specimens of mushrooms considered non-edible by local community members. The specimens belong to 45 genera; two from the phylum Ascomycota and 43 from Basidiomycota. With 15 families, Agaricales was the best represented order. The genera with the highest number of species were *Amanita* (12 spp.), *Cortinarius* (9 spp.), *Russula* (8 spp.), *Boletus,* and *Clitocybe* (5 spp.) (Additional file 1).

We identified the taxonomic species of 120 specimens, which corresponded to 100 mushroom taxa. From these, 26 are reported in the literature as edible, 20 as non-edible and 10 as toxic; edibility is unknown for the remaining species (Additional file 1). Among the species reported as edible, we found *Neoboletus* aff. *erythropus* (*hongo-rado*), *Hygrophoropsis aurantiaca* (*brindis*), *Clavariadelphus truncatus* (*bate*), and others (Additional file 1).

In both communities, 14 species were recognized as non-edible, and all non-edible mushrooms were considered poisonous. Species in such categories did not show a precise pattern with regard to their biological or ecological traits. An interesting finding was that, according to local people, edible ethnotaxa might become poisonous when they are too ripe. This is the case for *Amanita rubescens* (*mantecado de veneno*) and *Laccaria trichodermophora* (poisonous *xōlētl/xōlētl de veneno*). Others might become poisonous when the foraging season is over, for example, *Lyophyllum* gpo. *decastes* (mushroom of the bush/*hongo de mata/xolete*).

#### Identification attitudes

During our visits to the forests, we observed the different ways people approach non-edible mushrooms. There is a group of non-edible mushrooms that had proper names—*cītlal-nanacatl* and *hongo-rado—*that can easily be identified even from afar. These were handled with certain affinity, familiarity, and even pleasure. Nonetheless, people intentionally destroyed them as a warning, to indicate that they are poisonous and to prevent others from collecting it.

**Fig. 2:**
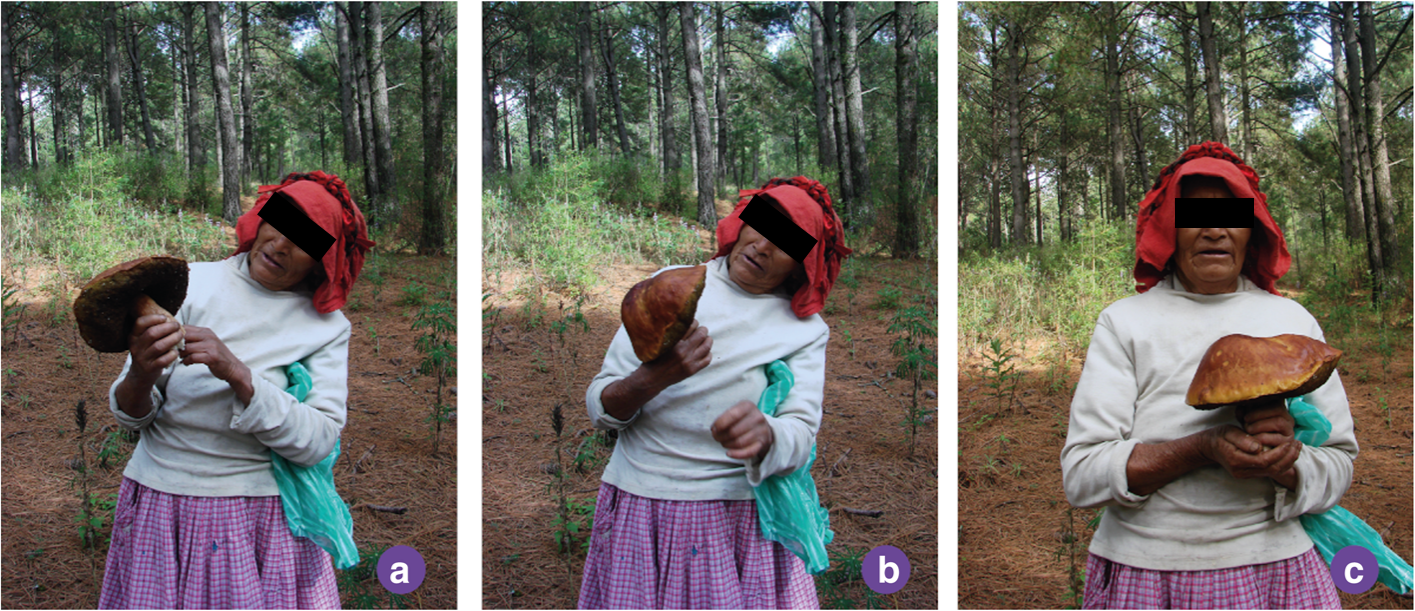
Non-edible mushroom discriminating process stages. **a** The specimen is carefully observed; one of its traits raises doubts. **b** Said trait is verified. **c** The specimen is confirmed as edible or rejected as inedible. Ms. Hermelinda holding a *xo-tomāh*

Another group of mushrooms did not have proper names (*camarón* and *i-tlatla in cuā-te-cax*). These were picked, such as teaching, joking, satisfying curiosity, and confirming their identity. They were smelled, tasted, observed, and then destroyed. The process of identification was executed in three stages: doubt, verification, and confirmation (Fig. 2).

The third group had no proper names and was never collected. Although we asked about these species, the local people clearly were indifferent to them and, therefore, they do no destroy them (*hongo venenoso*).

#### Local nomenclature for mushrooms

We obtained 179 local names (76 in FJM, 87 in SIBS, and 16 shared) for non-edible mushrooms, 29 in Nahuatl, 97 in Spanish, and 53 mixed (a combination of Nahuatl and Spanish). Traditional names corresponded to 130 genera, 47 taxonomic species, and two of superior orders. Local names did not correspond with scientific names in a 1:1 ratio; we therefore grouped the local names into ethnotaxa and identified the corresponding genus and species (Table 1). In general, mestizo Spanish speakers from FJM and Nahuatl speakers from SIBS designated the names of non-edible mushrooms by drawing comparisons with edible mushrooms. Local Spanish names were composed of two words, noun and a modifier, while Nahuatl names were formulated with a primary and a secondary lexeme.

**Table 1:** Nomenclature for non-edible mushroom ethnotaxa in the studied communities in Tlaxcala, Mexico

Ethnotaxon (names in Spanish, Nahuatl or both)		Scientific name
Francisco Javier Mina	San Isidro Buensuceso	
**Ajonjolinado**	***Cītlal-nanacatl*** Literal translation: star mushroom Idiomatic translation: star mushroom	*Amanita muscaria*
**Amantecado venenoso**	***Tōtol-te-nanacatl de veneno*** Literal translation: Turkey-rock-mushroom Idiomatic translation: poisonous turkey egg mushroom	*A. augusta*
**Amargo/amargoso**	***I-tlatla in caylita*** Literal translation: her-double-of-***caylita*** Idiomatic translation: ***caylita***’s twin	*Tricholoma virgatum*
**Enchilado malo**	***Chīl-nanacatl pitzō-nanacatl*** Literal translation: chili/red-mushroom rabies Idiomatic translation: pepper mushroom/red rabies mushroom	*Lactarius luculentus* *L. vinaceorufescens*
**Clavo/clavito malo**	Unknown	*Hygrocybe* sp. 1 *Lyophyllum* sp. 2
**Corneta de veneno**	***Tlapītzal de veneno*/** ***Tlapītzal veneno*so/*Tlapītzal*** **malo** Literal translation: (article) trumpet Idiomatic translation: poison/bad trumpet mushroom	*Phellodon niger* *Sarcodon* sp. 1
**Corneta de veneno/corneta venenosa-que no se come**	***Cuā-te-caxnanacatl de veneno/Cuā-te-caxnanacatl malo/Cuā-te-caxnanacatl que no se come*** Literal translation: head-rock-mortar-mushroom Idiomatic translation: poison/bad/inedible stone mortar (molcajete) mushroom	*Lactarius mexicanus L. smithii* *Russula densifolia*
**Escobeta de veneno/escobeta venenosa-mala**	***Xelhuāz nanacatl de veneno/Xelhuāz nanacatl malo*** Literal translation: (article) fork mushroom Idiomatic translation: poison/bad fork mushroom	*Clavulina* sp. 1 *Clavulina* sp. 2 *Ramaria abietina* *Ramaria gracilis*
Unknown	***Esquilon-nā-nanacatl de veneno*** Literal translation: bell-duplication-mushroom Idiomatic translation: poison bell mushroom	*Clitocybe odora*
**Hongo de los palos podridos de veneno**	Unknown	*Agrocybe* sp. 1 *Poliporoide* sp. 1 *Trametes* sp. 1
**Paloma**	Unknown	*Russula sancti-pauli*
**Pancita venenosa**	***Popozoh de veneno/Popozoh venenoso/Popozoh malo/Popozoh que no se come/Popozoh-rabia*** Literal translation: foam/venom Idiomatic translation: poisonous foam	*Suillus pseudobrevipes Suillus tomentosus*
**Pante de veneno** **Pante morado**	**Xo-tomāh de rabia, veneno, mal** **Xo-tomāh rabia** Literal translation: foot-fat Idiomatic translation: poisonous fat foot	*Baorangia* aff. *bicolor* *Neoboletus erythrophus* *Boletus* sp. 1 *Boletus* sp. 2 *Xerocomellus chrysenteron*
**Tecozah cimarrón** **Tecozah de veneno/ Tecozah venenosa** **Tlapaltecozah de veneno**	**Te-cōzah de pitzō-nanacatl /** **veneno/malo** Literal translation: rock-yello rabies Idiomatic translation: rabitic yellow stone mushroom	*Hygrophoropsis aurantiaca*
Unknown	***Xocoyolitlnanacatl malo, de veneno*** Literal translation: azadera mushroom Idiomatic translation: bad ***yerba azadera*** mushroom	*Cortinarius* sp. 6
Unknown	***Xōlētl de veneno*** Literal translation: type of mushroom Idiomatic translation: poisonous mushroom type	*Cortinarius* sp. 1; *Gymnopus* d*ryophilus* *Hygrocybe* sp. 1 *Leucopaxillus* sp. 1 *Lyophyllum decastes* group *Lyophyllum* sp. 2 *Pholiota* sp. 1 *Psathyrella* sp. 1

The primary lexeme was formed by a root or nuclear modifier that generally matched the name of the similar edible mushroom, or “edible lookalike.” The secondary lexeme or marginal modifier qualified the first and usually referred to the non-edibility attribute. In SIBS particularly, the secondary lexeme was composed of the relational noun *i-tlatla in*, which indicates kinship, and was followed by the term for the similar, edible counterpart, for example, *i-tlatla in tlalpīltzal*.

There was, however, a contrast in the nomenclature for the most culturally important non-edible mushrooms. These had a proper name that did not refer to an edible lookalike, even if one exists. Such is the case for *Amanita muscaria* (*cītlal-nanacatl/ajonjolinado*) and *Neoboletus* aff. *erythropus* (*hongo-rado*) in FJM. These proper names for non-edible species may contain modifiers that indicated specific traits that act as differentiators between varieties, for example, *ajonjolinado blanco de encino*, *cītlal-nanacatl blanco de oyamel*. Up to 40% of local names were mentioned by at least four people, and in general, these names were descriptive (*cuerudo*, *volcancito*, *ruleta*, *vidrioso*, etc.) references to elements of the environment, such as animals (*uña de ratón*, *venadito*, *pipilo*), or references to characteristics (*señoritas*, *oreja de diablo*, etc.).

#### Dual worldview

People from both the communities we studied conceptualized non-edible mushrooms as existing in conjunction with a similar edible mushroom, and this concept emanated from a perception of the duality between good (edible) and bad (poisonous). Local names were a clear example of such dual perception; in fact, people declared that to know an edible mushroom, it is necessary to know its poisonous counterpart. This helps prevent mistakes that would put one’s health at risk. In both communities, people thought that the toxic element is evident in the mushrooms’ morphology: “Edible and poisonous mushrooms are very similar at first, sight, but if you know them well, you can see the difference between them.”

**Fig. 3:**
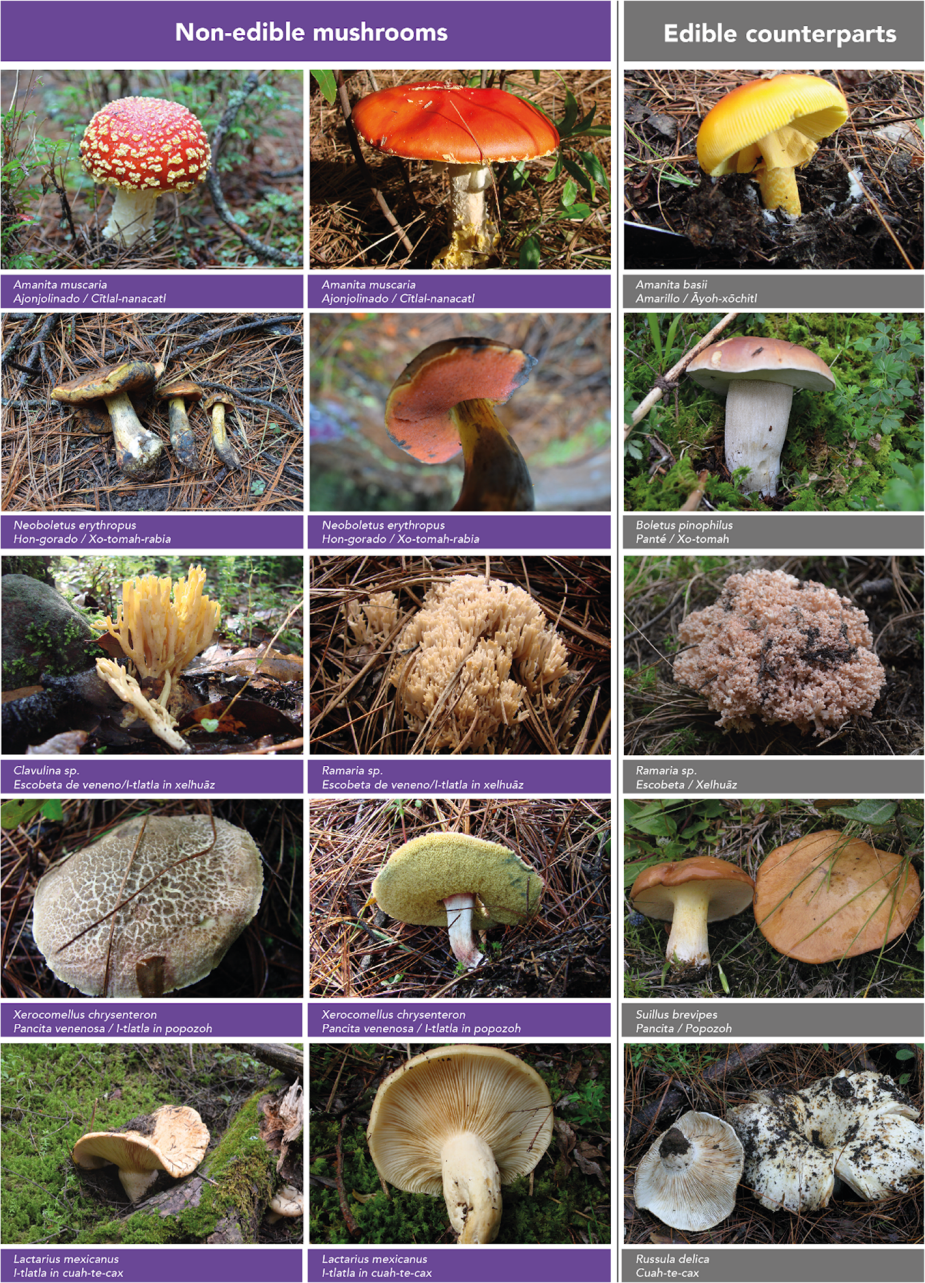
Examples of edible and non-edible mushroom duality

Figure 3 shows the dual perception of non-edible mushrooms mentioned by more than 10% of people from both communities. For members of the FJM and SIBS community, the relationship between edible and non-edible mushrooms was very narrow and they even considered that one cannot exist without the other. This is evidence of a line of thought that maintains that there is the balance in the universe; there cannot be more bad than good, since good controls bad and vice versa.

In FJM, respondents even compared the duality of good and bad mushrooms to the birth of twins; where according to their beliefs, one will get the good attributes while the other gets the bad ones. In SIBS, people used the noun *i-tlatla in* meaning “the lookalike” to refer to the good and bad duality.

#### Identification criteria

In total, interviewees mentioned 101 identification criteria for non-edible ethnotaxa, which were grouped as 32 general and 87 specific criteria. Both studied communities based their identification on 34 central criteria (abundance, unpleasant appearance, change of color after rough treatment, gills, fruitbody, color of the stipe and pileus, consistency, shape and thickness of the fruitbody, absence of worms, hymenium shape, pileus ornamentation, taste, and surface area). These were the most informative, detailed, and specific criteria, which sometimes were included in the traditional local mushroom names. Conversely, many specific criteria were used exclusively in only one of the two communities, 24 in FJM (for example, big loculus, flat pileus and partial veil) and 43 in SIBS (changing color with maturation, hymenium color, leathery cuticle, small stipe, pileus size and thickness, and fast decomposition).

**Fig. 4:**
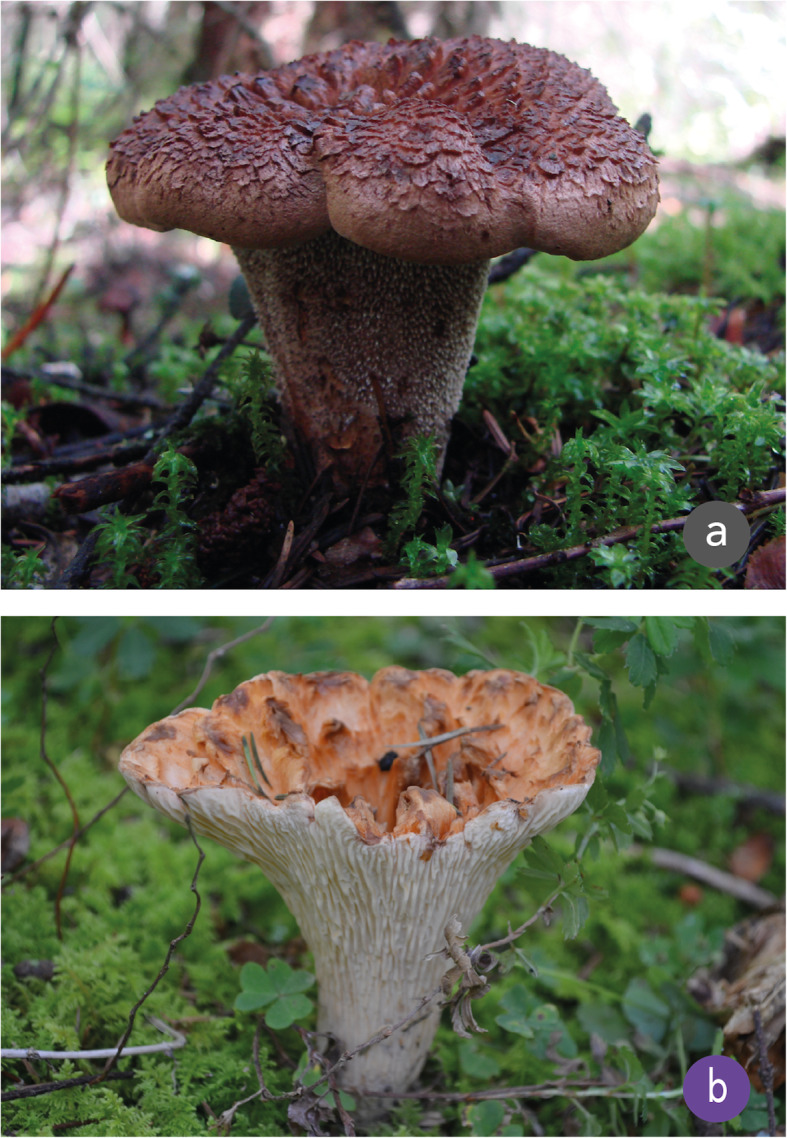
Identification of a non-edible mushroom by comparison with a similar edible counterpart. **a** The *Phellodon niger*-*Sarcodon* sp. (in Nahuatl, *I-tlatla in tlapītzal*), is recognized by its unpleasant appearance, namely a distinct mix of colors (black, brown and purple), a dentate hymenium (“the underside is like a little brush”) and very flaky scales (“the top of the cap doesn't look smooth, like it's rolling up”). **b** *Tlapītzal* (*T. floccosus*) One of the most appreciated mushrooms in the San Isidro Buensuceso (SIBS) community

These identification criteria are evidence that traditional methods for mushroom identification are based on the traits of fresh mushroom, while some attributes such as color, size, texture, thickness, weight, smell, taste, consistency, and biotic environment, among others, are essential to differentiate edible from non-edible mushrooms. These criteria are related to the main characteristics that are used in classic taxonomy to distinguish organisms at a generic level, for example, scales on the pileus for the genus *Amanita;* color change after rough treatment for Boletales; branching patterns for coralloids; dentate hymenium for *Sarcodon* and *Phellodon* and sporome color to discriminate varieties (Fig. 4).

In both villages, we observed a consensus regarding the specific identification criteria for the most culturally important non-edible mushrooms (*ajonjolinado*-*cītlal-nanacatl*, *pancita venenosa*-*popozoh-rrabia*, *panté de veneno*-*xo-tomāh de veneno*, *escobeta de veneno*-*xelhuāz de veneno*, *corneta de veneno*-*tlalpīltzal de veneno*). Meanwhile, for ethnotaxa of lesser importance, the less relevant they were, the less precise were the criteria, and there was no consensus.

#### Classification systems

**Fig. 5:**
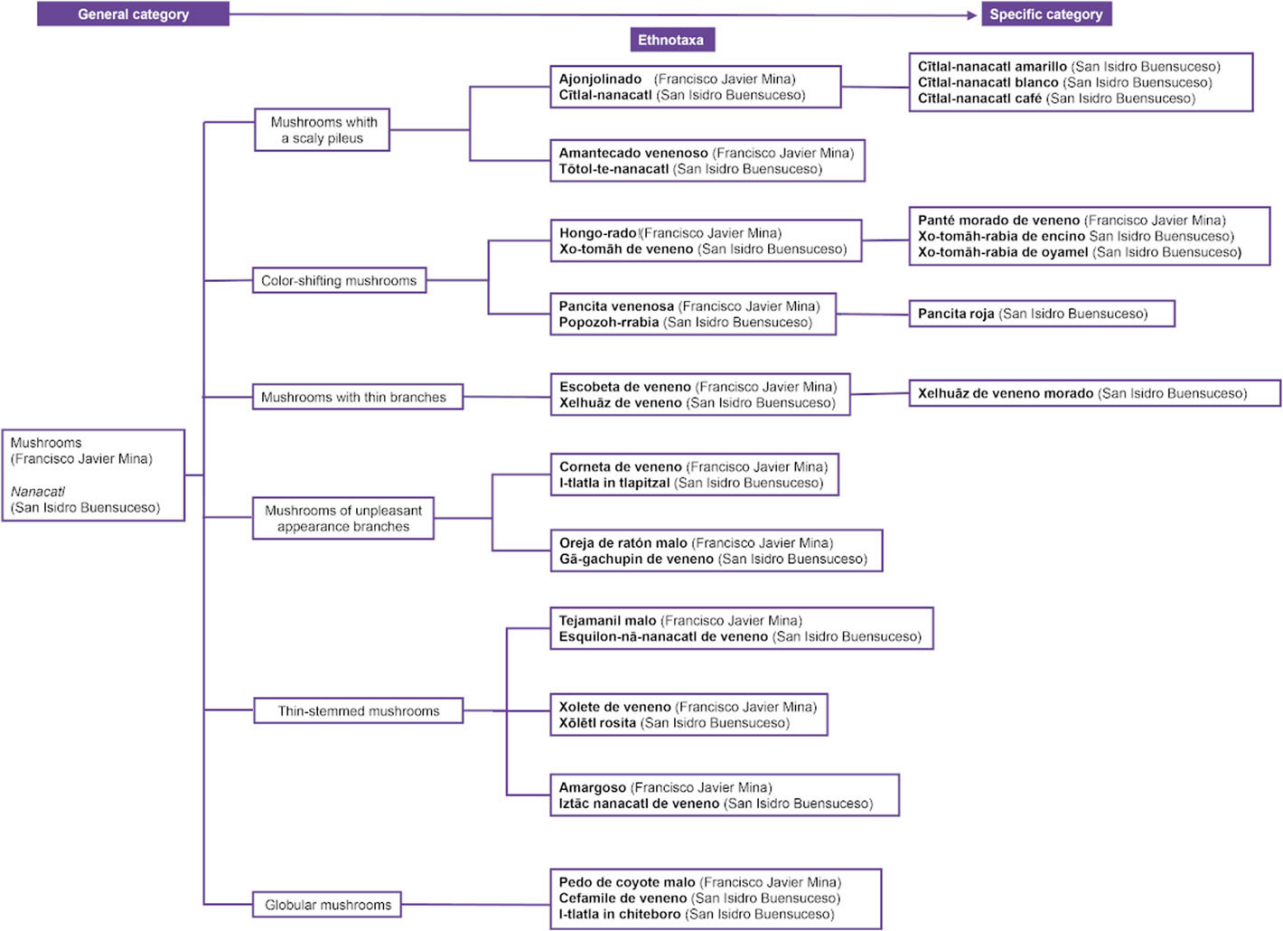
Hierarchical taxonomy of non-edible mushrooms

In both communities, we identified a classification system that is based on anthropocentric utility. It established a general group, mushrooms/*hongos*/*nanacatl*, which was subdivided into two subgroups: (1) edible mushrooms/*hongos comestibles*/*cualinanacatl* (encompassing all mushrooms that are part of their diets), and (2) poisonous mushrooms/*hongos venenosos*/*pitzō-nanacatl* (encompassing all non-edible mushrooms or those of unknown edibility). However, both the lexical aspects of local names, and the identification criteria both referred to traits that can be classified into more complex subdivisions (Fig. 5). This classification proposal corresponded to a hierarchical inclusion scheme that is based on the structural criteria for morphological identification (shape, color, habitat, substrate, smell, taste, consistency), to distinguish non-edible species from edible species.

#### Uses

In both communities, most people (55.5% in FJM and 66.6% in SIBS) stated that non-edible mushrooms are useless. However, an important proportion of the population mentioned different anthropocentric categories, such as medicine (18.5% in FJM and 11.1% in SIBS), drugs (3.8% in FJM and 3.7% in SIBS), and insecticide (3.8% in FJM and 11.1% in SIBS). Using mushrooms as insecticide was the most common in SIBS, and it was attributed to *A. muscaria*, which was also recognized as a medicine when consumed in small amounts to induce vomiting (current use) or to control tachycardia (discontinued use). This species was also reported as edible, provided the scales and cuticle are removed. Medicinal and culinary applications of *A. muscaria* have disappeared, because it was basically the elders that used to use the mushrooms for such purposes.

In FJM, 25.6% of interviewees mentioned that *Neoboletus* aff. *erythropus* (*hongo-rado*) is traded in the main markets of Puebla and Mexico City. The sale of this mushroom is recent, and the mushroom pickers/*hongueros* had to come to terms with the idea that this mushroom is being used as a medicine in both cities before they would agree to sell a specimen they had considered to be poisonous. Currently, there are families that specialize in collecting this species, and they reap significant economic benefits from collecting several kilograms a day. Nonetheless, the mushroom’s medicinal use has not yet been integrated into traditional practices in FJM, since the people there still consider it to be a poisonous mushroom, but with commercial applications.

#### Symptoms and local remedies used to treat mycetism

**Fig. 6:**
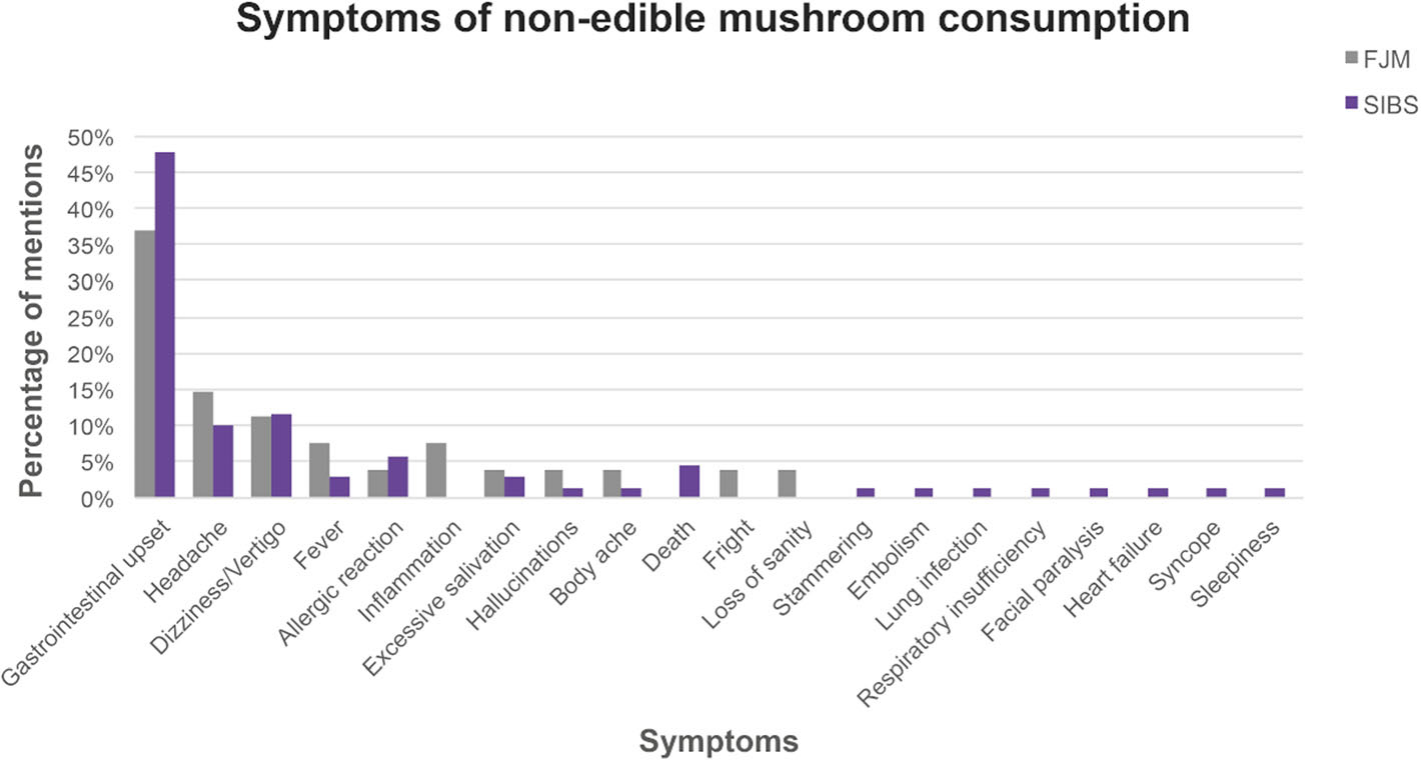
Non-edible mushroom consumption symptoms, by percentage of mentions

While most members of both communities associated the consumption of non-edible mushrooms with death (49% in FJM and 59% in SIBS), they acknowledged that some of these mushrooms cause only specific intoxications (38% FJM and 30% SIBS), hallucinations (3% FJM), and even cancer (1% FJM and SIBS). Interviewees noted that intoxications are not all the same, but that every mushroom has a distinctive type of poison and, consequently, that the symptoms following consumption are specific, ranging from gastrointestinal problems to death (Fig. 6).

In the two communities, a significant proportion of the interviewees indicated that there are not any existing local remedies for intoxications caused by wild mushroom consumption (20% in FJM and 49.2% in SIBS). They considered that the only option is to consult a physician to get appropriate medication. On the other hand, 32% of the people interviewed in FJM and 31.6% in SIBS said that there were local remedies that could mitigate the discomfort caused by intoxications and even help to prevent death.

There are, therefore, traditional remedies aimed at mitigating the symptoms caused by mushroom intoxication. For example, the treatment for gastrointestinal problems is to drink a combination of garlic tea, vinegar, and milk to purge the person of the ingested mushroom, eliminating the effects of the intoxication. This remedy is followed by an infusion of peppermint, chamomile, and skunk *epazote* (*Chenopodium ambrosioides* L.) to help to relieve stomach discomfort. Interestingly, SIBS people mentioned that ingestion of alcoholic beverages, such as *pulque* (fermented sap of some *Agave* species) and *aguardiente* (cane liquor), is one of the most effective cures to avoid death by mushroom poisoning.

In both sites, this knowledge of mushrooms was distributed within the adult population and was gender related. Women are the ones who go to the forest to forage for resources and medicinal plants, while men—who devote their days to agriculture—show a more detailed knowledge of the remedies for the symptoms of intoxication. Such knowledge is of great value and has contributed to the fact that poisoning remains low in the area; there are years in which no poisoning case has been recorded.

#### Cultural importance

Cultural importance indicators showed that the most important ethnotaxa for both communities were *Amanita muscaria* (*ajonjolinado-cītlal-nanacatl*), *N.* aff. *erythropus*-*Xerocomellus chrysenteron* (*hongo-rado*-*panté*, *venenoso-xo-tomāh rabia*), and *Suillus pseudobrevipes*/*S. tomentosus* (*popozoh venenoso*, *de veneno*). When comparing the taxa used in both villages, in FJM, *N.* aff. *erythropus* (*hongo-rado*) was a different ethnotaxa than the rest of Boletales species while in SIBS this species was considered to be a part of this group. For statistical analysis, *N.* aff. *erythropus* was placed into Boletales (Table 2).

**Table 2:** Cultural importance of the non-edible mushrooms present in studied communities in Tlaxcala, Mexico

Scientific names	Local name (in Nahuatl, Spanish or both)	Mention frequency		Mention order		
		NM	% M	NVOM	ROV	MMO
*Amanita muscaria*	***Cītlal-nanacatl ajonjolinado***	65	71.43%	44 (1st place) 11 (2nd place) 3 (3rd place) 4 (4th place) 2 (5th place) 1 (7th place)	52.04	4.32
*Neoboletus erythropus* *Xerocomellus chrysenteron*	***Hongo-rado*** ***Panté venenoso xo-tomāh rabia***	59	64.83%	18 (1st place) 17 (2nd place) 9 (3rd place) 9 (4th place) 4 (5th place) 1 (6th place) 1 (15th place)	32.73	5.23
*Suillus pseudobrevipes* *S. tomentosus*	***Popozoh venenoso*** ***Popozoh de veneno***	24	26.37%	3 (1st place) 6 (2nd place) 7 (3rd place) 1 (4th place) 2 (5th place) 2 (6th place) 1 (7th place) 2 (8th place)	9.71	9.09
*Clavulina* sp. 1 y sp. 2 *Ramaria abietina* *R. gracilis*	***Escobeta de veneno*** ***Xelhuāz Nanacatl de veneno***	23	25.27%	1 (1st place) 3 (3rd place) 6 (4th place) 5 (5th place) 2 (6th place) 2 (7th place) 1 (8th place) 1 (9th place) 1 (12th place) 1 (14th place)	5.62	9.58
*Lactarius mexicanus*	**Corneta de veneno,** **Cuā-te-caxnanacatl de veneno**	17	18.68%	3 (2nd place) 3 (3rd place) 6 (4th place) 1 (6th place) 2 (7th place) 1 (10th place) 1 (12th place)	4.64	9.75
*Amanita* aff. *cinereoconia* *A. xylinivolva* *Lyophyllum* sp. 2	**Hongo blanco venenoso** **Iztāc nanacatl de veneno**	13	14.29	2 (2nd place) 2 (4th place) 3 (5th place) 3 (6th place) 2 (7th place) 1 (10th place)	2.99	10.24
*Lactarius vinaceorufescens*	**Enchilado malo** **Chīl-nanacatl de pitzō-nanacatl, Chīl-nanacatl de veneno**	10	10.99	1 (1st place) 1 (3rd place) 3 (4th place) 1 (6th place) 2 (7th place) 1 (13th place) 1 (20th place)	2.66	10.42

*NM* number of mentions, *% M* proportion of mention, *NV OM* number of times mentioned in each order of mention, *OVR* ordinal value of rank, *MMO* mean order of mention

In FJM, the most important taxa were *A. muscaria* (*ajonjolinado de veneno*), *Pholiota* sp. 1/*Psathyrella* sp. 1 (*xolete de veneno*), and *N.* aff. *erythropus* (*hongo-rado*). The most frequently mentioned was *A. muscaria* (*ajonjolinado de veneno*) with a clear difference between this species and the remaining ones (Additional file 2). In SIBS, Boletales (*xo-tomāh de veneno*), *A. muscaria* (*cītlal-nanacatl*), and *Sarcodon* spp./*Phellodon* spp. (*tlalpīltzal de veneno*) were the most commonly cited. Although, in JM, the Boletales order was rated the highest, *A. muscaria* (*cītlal-nanacatl*) was mentioned first more often, explaining its higher ordinal value of rank (Additional file 3). Also, there was no consensus between both communities on the status of the two most important taxa (*A. muscaria* in FJM, and *Baorangia* aff. *bicolor*, *N.* aff. *erythropus*, and *Xerocomellus chrysenteron* in SIBS).

## Discussion

Our results revealed that the traditional knowledge of non-edible mushrooms is vast and profound, providing proof of their importance in the people’s worldview. We described the characteristics used to identify toxic mushrooms and to distinguish what their utility is. In such sense, we propose that the use of wild mushrooms is not a central axis for building knowledge around them. According to Berlin [8], knowledge is built from the identification of attributes that play a role in the culture and, according to Lévi-Strauss’ structuralism [6], comprehension comes first—before a utility is assigned.

### Traditional knowledge of non-edible mushrooms

The use of wild mushrooms is possible thanks to the lore accumulated in our cultural memory, based on our worldview. Amassing such a cultural heritage is helped by the recognition of distinctive characteristics that allow people to differentiate non-edible mushrooms from edible ones. Conceptual representations are built from group and individual experiences that become rational, collective knowledge and remain in the collective unconscious [73].

Our data showed that non-edible fungi species are key factors in the *kosmos*, *corpus*, and *praxis* of traditional knowledge of wild mushrooms [5]. We identified more than 100 non-edible taxa; in contrast, other ethnomycological studies usually mention up to 17 species that are recognized as toxic or poisonous [13, 40, 74]. Moreover, the characterization of local knowledge about non-edible mushrooms has been superficial and is generally the result of generalizations about edible mushrooms [11, 16, 19, 75].

It is interesting that, in this study, among the species recognized as non-edible, some have previously been reported as edible [26], such as *Amanita rubescens* and *Laccaria trichodermophora*, which were considered toxic when very ripe. This concurs with studies on the Karbi people of Northeastern India that found that some species lose their taste when ripened and are, therefore, collected and consumed only in their juvenile stage [17]. This phenomenon has also been recorded in Southern Mexico, where the Tseltales report some edible species as toxic [13]. Similarly, the consumption of species known as toxic in other parts of the world, such as *Tricholoma equestre*, has been documented [76]. In the present study, in both communities, the local names *blanco venenoso* and *iztāc nanacatl de veneno* refer to white species of the genus *Amanita* that have already caused intoxications in the region [26]. However, we did not find any of their fruitbodies to make a proper taxonomic identification.

**Fig. 7:**
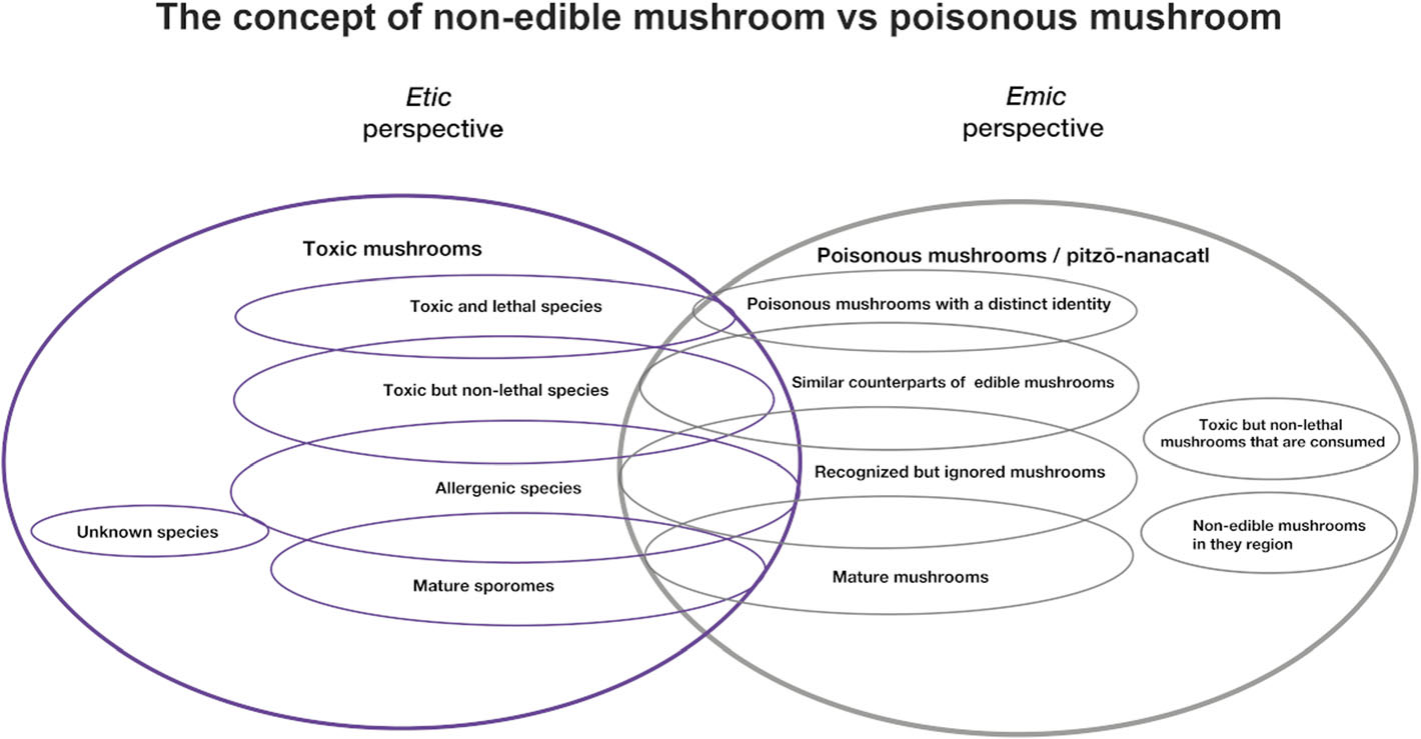
Representation of the concept of non-edibility in mushrooms from the perspective of *etic vs emic*

The fact that people considered as poisonous all mushrooms that they do not include in their diet indicates that knowledge and interest depends on traditions of consumption [15, 17, 19, 30, 39, 40]. There are only a few cases where a species that has not been used within the family nucleus has been integrated into the diet, showing a degree of rejection of the unknown, due to intoxication risks [77]. In contrast, mushroom pickers and traders were more willing to learn about species that are not part of their lore. In Fig. 7, we show a graphic contrast of the non-edible mushroom concepts using the *emic versus etic* approaches, evidencing that the general category “poisonous mushrooms” can only be explained from an *etic* perspective [78].

The way people identify non-edible mushrooms during the collection process is a practical depiction of their knowledge: those more similar to edible species are more important and appreciated. General identification criteria matched with those reported in ethnomycological research conducted in the highlands of Mexico [16]. This sustains the findings by Estrada-Torres and Aroche [79], Montoya et al. [26], and Moreno-Fuentes [80]: Inhabitants of highland zones possess exhaustive knowledge to differentiate toxic species from edible ones, and they even learned to recognize traits and establish traditional criteria for mushroom recognition. Such precision in identifying species has also been reported for the Kurya tribe in Tanzania and the Karbi culture in India, who use traditional indicators associated with specific characteristics of the mushrooms that grow in their territory, for example, smell, color, size, habitat, and substrate where they grow [17, 75]. Conversely, cultures from The Northwestern Andes in Colombia use strong and vivid colors, spicy or bitter taste as hazard indicators [39]. Some of the criteria used by cultures resemble those used in classic mycology [17].

### Nomenclature and discrimination of non-edible mushrooms

Local names are a collective mental construct, where each ethnotaxon is delimited by perceptible characteristics surrounded by the particular wisdom of every culture [73]. The 181 local names referring to non-edible mushrooms obtained in our study showed the wealth of knowledge that exists on the subject; this number represents 60% of all traditional names of non-edible mushrooms currently recognized in Latin America [81]. On a regional scale, our findings represent more than 10 times the number of local names previously reported in the area [26, 47]. Despite the vast diversity of local names, only 13 in FJM and 15 in SIBS were mentioned by more than 10% of the interviewed participants. A significant number of local names (40.2%) was idiosyncratic [3].

Studies conducted in different parts of the world report that non-edible mushrooms are identified in general terms, without specific names, and only classified as non-edible [13–15, 20, 40]. A few studies report one or two local names to refer to non-edible ethnotaxa [2, 11, 26, 30, 82]. One example is the area of Masovia, Poland, where the mean number of listed inedible or poisonous fungi taxa was 1.7, with a maximum of six [74]. The names emerge by contrasting non-edible with edible mushrooms, and each ethnotaxon is delimited by certain types of characteristics that allow for its recognition. In this nomenclatural scheme, we observed that only the most remarkable species, or the ones that represent health hazards, have a name, but as their relevance declines, so does the interest in giving them names [9, 11, 13, 30, 40]. In addition, the names demonstrate the pragmatic character of the nomenclature, where a vast knowledge of morphologic, ecologic, phenologic, and qualitative traits is employed to allow the recognition of every ethnotaxon [8].

### Dual worldview

Traditional mycological knowledge is developed and acquired in an integral manner. Those who wish to learn about mushrooms cannot limit themselves to only the useful ones, they also need specific information to be able to differentiate them from toxic lookalikes [41]. This reflects the close relationship between edible and non-edible mushrooms and shows their dual nature. The “double edible” approach has been reported in diverse ethnomycological studies, which stipulate that the names of non-edible mushrooms must contain terms that indicate the opposition to an edible variety, e.g., the bad, partner, older brother, friend [13, 30]. Haro-Luna [19] documented the dual worldview of the Wixarika people in Mexico regarding edible *vs* toxic mushrooms: the first pertains to tangible reality, while the second pertains to the spiritual realm and must not be eaten. Dual perception of mushrooms is not restricted to edible/non-edible organisms; for example, the Mixes and the Mazatecos point out that one of the rules for consuming sacred mushrooms is to ingest them in pairs, symbolizing the balance of the universe and the duality of woman and man [83, 84].

### Classification of non-edible mushrooms

Mushroom categorization is based on assigned use [20]. Identification criteria have a hierarchical structure that is based on observable specific traits [8, 9, 11], where utilization is signified in the second term, indicating that people classify and use resources at the same time [85, 86]. Categories are constructed from judgments of similarity or hierarchical resemblance networks, linguistically represented by descriptive terms that aim to represent nature in an orderly manner [11].

### Poisonous mushrooms with other uses

Different uses for non-edible mushrooms have been recorded in the region of *La Malintzi* National Park [26, 47, 87]. The use of *A. muscaria* as an insecticide has been highly documented, and in fact, many of its local names refer to its link with flies [15, 87]. Its medicinal use has also been recorded in different Mexican communities of Mexico and the world [14, 15, 74, 87]. This use is closely related to its appearance in rituals, given the species is a sacred element in diverse cultures, mainly in Europe and Asia [88]. As well, edibility of this species is frequently cited in some communities from highland zones [15, 88].

In the case of *N.* aff. *erythropus* (*hongo-rado*) and its use as medicine, this is a recent phenomenon derived from commercial interests [26]. Association of non-edible mushrooms to neurotropics (*emborrachantes* or *locos*) is a generalized perception, perhaps because they have sacred uses in diverse cultures around the world, and such uses are related to their psychoactive properties [15, 16, 30].

### Symptoms and local remedies to treat mycetism

Members of both studied communities associated the consumption of poisonous mushrooms with death; however, they also admitted that some species cause only discomfort or intoxication. Some studies suggest that the recognition of adverse symptoms is a part of traditional mycological knowledge, specifically, the detection of diverse types of mycetism [16]. However, there are some exceptions. For instance, Mayan people from Chiapas describe a general pattern: The first stage is a psychoactive intoxication, followed by gastrointestinal disorders, tissue, and organ damage, and finally, death [30]. Our results show the opposite of this particular belief, since people participating in our study claimed that not all types of mushroom intoxications are equal, because each mushroom has its own specific poison. Consequently, the symptoms provoked are specific and not all are fatal.

In fact, there are several types of local remedies, which provide evidence of the richness of local knowledge about non-edible mushrooms. For instance, in Northeast India, the Karbis use 12 different traditional cures for mycetisms, e.g., corn, citric, rice, tamarind, soil, and human feces, among others [17]. Various studies in Central and Southern Mexico have reported that the most prevalent traditional remedies, those with laxative effects, such as salted water, crushed garlic, cooking oil, lemon juice, or mezcal [16]. This shows some degree of convergence of local knowledge from different regions.

### Non-edible mushroom’s cultural importance

**Fig. 8:**
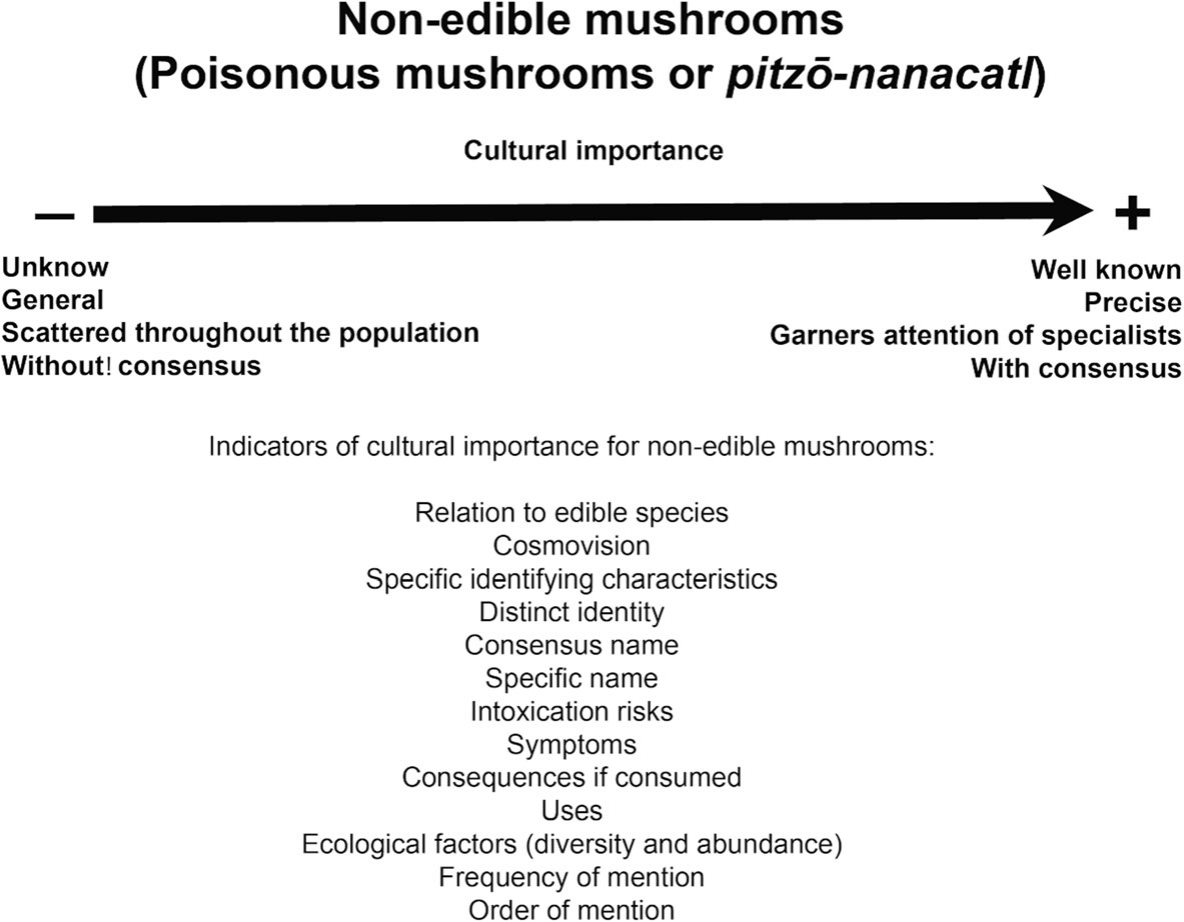
Non-edible mushrooms cultural importance indicators

We observed that traditional knowledge about non-edible mushrooms is scarce and only its most relevant elements are distributed throughout most of the community. Knowledge about these mushrooms is evidence, however, that some indicators can be used to evaluate their role in a culture [89]. In general, the cultural importance of non-edible mushrooms resulted from the knowledge of the entire range of species, which led to different uses (Fig. 8) and, although non-edible mushrooms do not have explicit uses, they play relevant roles in many cultures [40].

The most important non-edible mushrooms of both communities were those that are most similar to the most important edible mushrooms. Such trend was also reported in two Mayan groups in Chiapas, Mexico: the Tsotsiles and the Tseltales [13, 40], but also it was reported for people interviewed in Mazovia, Poland [74]. The relevance is determined by the role of the double edible, according to specific categories: (1) those present in people’s worldview through a dual scheme (good/bad), and in their stories (myths, legends and tales); (2) those related to an edible duality but with their own identity, which separates them from the rest in a nomenclatural form; (3) those with a set of clear and precise traits that group them in specific ethnotaxon and that have consensus names; (4) those whose consumption has very well-known symptoms and consequences, (5) those known to be toxic, but used anyway; (6) the most abundant in the forest; and (7) the most frequently mentioned in free listings.

Based on our information, this is the first study attempting to deepen the description of traditional knowledge regarding non-edible mushrooms using a combination of qualitative and simple quantitative indicators [26, 32] to better understand their role in a given community. This is the initial step to propose the use of composite indexes [35, 37] or specific methods to obtain even more robust information in order to comprehend the cultural relevance of this residual category of organisms.

## Conclusions

Local knowledge about non-edible mushrooms is a fundamental part of traditional mycological knowledge. In Mesoamerica, this comprehension is constructed based on a dual principle, essential for discriminating potentially dangerous mushrooms. Non-edible mushrooms form a very large and heterogeneous group, which is defined by their comparison with edible mushrooms; in other words, all that is non-edible is poisonous. Preferences for certain species result from sociocultural factors as well as from beliefs that emerge and are transmitted through myths, legends, and taboos, which are specific to every community and culture.

Previously, resources having no utility were considered as not important for cultures and, therefore, were neither studied nor named. Our study, however, documented a huge amount of local knowledge about regarding non-edible mushrooms or mushrooms that are not used for anything. Such knowledge is scattered, rarely leads to consensus or lexical retention, is narrowly related to edible mushrooms, and is essential to avoid mycetism and death. Local knowledge of non-edible mushrooms depends on the status of each mushroom in the cultural domain, which is homogeneous, precise, profound, and with consensus for the most important species. But, as the relevance of the species diminishes, knowledge about them becomes heterogeneous, very general, dispersed, and lacking consensus.

Documentation, diffusion, and enhancement of traditional knowledge about non-edible mushrooms comprise an encouraging strategy to prevent intoxications. To guarantee that wild mushroom consumption is practiced in a safe manner, it is necessary to apply local knowledge during foraging and preparation activities, and in the administration of primary care to counter intoxications.

## Supplementary Information

**Additional file 1.** List of taxa recognized as non-edible

**Additional file 2.** Cultural importance of non-edible mushrooms in Francisco Javier Mina

**Additional file 3.** Cultural importance of non-edible mushrooms in San Isidro Buensuceso

## Data Availability

Data sharing is not applicable to this article as no data sets were generated or analyzed during the current study.

## Abbreviations

FJM: Francisco Javier Mina
SIBS: San Isidro Buensuceso
PNLM: La Malintzi National Park
MF: Mention frequency
MO: Mention order
ROV: Rank ordinal value
MMO: Mention mean order

## References

[1] Ford RI, Anderson E, Pearsall D, Eugene Hunn NT (2011). History of ethnobiology. Ethnobiology.

[2] Agnihotri S, Si A (2012). Solega ethno-ornithology. J Ethnobiol..

[3] Zamudio F, Hilgert NI (2015). Multi-dimensionality and variability in folk classification of stingless bees (Apidae: Meliponini). J Ethnobiol Ethnomed.

[4] Zent SZ, Zent E, Urbani B, Lizarralde M (2020). Co-ecology of Jotï, primates, and other people: a multispecies ethnography in the Venezuelan Guayana. Neotrop Ethnoprimatology Indig peoples’ perceptions of and interactions with nonhuman primates.

[5] Toledo VM. Indigenous peoples and biodiversity. Encycl Biodivers. 2001:452–63 10.1016/B978-0-12-384719-5.00299-9.

[6] Lévi-Strauss C (1968). Mitológicas I: Lo crudo y lo cocido.

[7] Berlin B, Breedlove DE, Raven HP (1973). General principles of classification and nomenclature in folk biology. Am Anthropol.

[8] Berlin B (1992). Ethnobiological classification: principles of categorization of plants and animals in traditional societies.

[9] Hunn E (1982). The utilitarian factor in folk biological classification. Am Anthropol.

[10] Lampman AM (2007). General principles of ethnomycological classification among Tseltales in Tenejapa Chiapas. J Ethnobiol.

[11] Ellen R (2008). Ethnomycology among the Nuaulu of the Moluccas: putting Berlin’s “general principles” of ethnobiological classification to the test. Econ Bot.

[12] Zent E, Zent S (2016). Ebojto: Plantas Trepadoras entre los jotï, Guayana Venezolana. Etnobiología.

[13] Ruan-Soto F. Highly cultural significant edible and toxic mushrooms among the Tseltal from the Highlands of Chiapas, Mexico. Ethnobiol Conserv. 2020;32:1–20. https://ethnobioconservation.com/index.php/ebc/article/view/414. 10.15451/ec2020-08-9.32-1-20.

[14] Boa E (2005). Los hongos silvestres comestibles: Perspectiva global de su uso e importancia para la población. Prod. For. no madereros.

[15] Yamin-Pasternak S, Pearsall D, Eugene Hunn NT, Anderson E (2011). Ethnomycology: fungi and mushrooms in cultural entanglements. Ethnobiology.

[16] Ramírez-Terrazo A, Montoya A, Caballero J, Moreno-Fuentes A, Garibay-Orijel R (2014). Una mirada al conocimiento tradicional sobre los hongos tóxicos en México. La etnomicología en México, estado del arte.

[17] Prila y Teron RH (2018). Eschewing poisons: an ingenious wisdom of foraging macrofungi by Karbi ethnic group in North East India. Curr Sci.

[18] Ruan-Soto F. Intoxicaciones por consumo de hongos silvestres entre los tsotsiles de Chamula, Chiapas, México. Soc y Ambient. 2018;7. scielo.org.mx/scielo.php?script=sci_arttext&pid=S2007-65762018000200007.

[19] Haro-Luna MX, Ruan-Soto F, Guzmán-Dávalos L (2019). Traditional knowledge, uses, and perceptions of mushrooms among the Wixaritari and mestizos of Villa Guerrero, Jalisco, Mexico. IMA Fungus.

[20] Reyes-López RC, Montoya A, Kong A, Cruz-Campuzano EA, Caballero-Nieto J (2020). Folk classification of wild mushrooms from San Isidro Buensuceso, Tlaxcala, Central Mexico. J Ethnobiol Ethnomed.

[21] Cano-Estrada A, Romero-Bautista L (2016). Valor económico , nutricional y medicinal de hongos comestibles silvestres Economic, nutritional and medicinal value of edible wild mushrooms. Rev Chil Nutr.

[22] Arora D. California porcini: three new taxa, observations on their harvest, and the tragedy of no commons. Econ Bot. 2008;62(3):356–75 https://link.springer.com/article/10.1007%2Fs12231-008-9050-7. 10.1007/s12231-008-9050-7.

[23] Trutmann P, Unsaac MEH, Unsaac AQ, Luque A (2012). Native mushrooms, local knowledge, and potential for food and health in the Peruvian Andes: update 2012.

[24] Pérez-Moreno J, Martínez-Reyes M, Yescas-Pérez A, Delgado-Alvarado A, Xoconostle-Cázares B (2008). Wild mushroom markets in central Mexico and a case study at Ozumba. Econ Bot.

[25] Guzmán G (2011). El Uso Tradicional de los Hongos Sagrados: Pasado y Presente. Etnobiología..

[26] Montoya A, Hernández-Totomoch O, Estrada-Torres A, Kong A, Caballero J (2003). Traditional knowledge about mushrooms in a Nahua community in the state of Tlaxcala, México. Mycologia.

[27] Ruan-Soto F, Mariaca R, Alvarado R (2011). Intoxicaciones mortales por consumo de hongos: una cadena de errores. Eco Front Rev cuatrimestralde Divulg la Cienc.

[28] Shepard G. The forgotten kingdom: Mushrooms and ethnobiology. Paper presented at the American Anthropological Association Meetings, Atlanta GA. Manuscript. p. 16.

[29] Shepard GH, Arora D, Lampman A (2008). The grace of the flood: classification and use of wild mushrooms among the highland Maya of Chiapas. Econ Bot.

[30] Zent EL, Zent S, Iturriaga T (2004). Knowledge and use of fungi by a mycophilic society of the Venezuelan Amazon. Econ Bot.

[31] Teke NA, Kinge TR, Bechem E, Nji TM, Ndam LM, Mih AM (2018). Ethnomycological study in the Kilum-Ijim mountain forest, Northwest Region, Cameroon. J Ethnobiol Ethnomed.

[32] Montoya A, Kong A, Torres-García E, Moreno-Fuentes A, Garibay-Orijel R (2014). Síntesis de los métodos cuantitativos empleados en Etnomicología. La Etnomicología en México Estado del Arte.

[33] Burrola-Aguilar C, Montiel O, Garibay-Orijel R, Ziumbo-Villarreal L (2012). Conocimiento tradicional y aprovechamiento de los hongos comestibles silvestres en la región de Amanalco, Estado de México. Rev Mex Micol.

[34] Montoya A, Torres-García EA, Kong A, Estrada-Torres A, Caballero J (2012). Gender differences and regionalization of the cultural significance of wild mushrooms around la Malinche Volcano, Tlaxcala, Mexico. Mycologia..

[35] Garibay-Orijel R, Caballero J, Estrada-Torres A, Cifuentes J (2007). Understanding cultural significance, the edible mushrooms case. J Ethnobiol Ethnomed.

[36] Bautista-Nava E, Moreno-Fuentes A, Pulido-Silva MT, Valadez Azúa R, Moreno-Fuentes A, Pulido-Silva T, Mariaca-Méndez R, Valadéz-Azúa R, Mejía-Correa P, Gutiérrez-Santillán TV (2010). Bases bioculturales para el aprovechamiento y conservación de los hongos silvestres comestibles en el Municipio de Tenango de Doria, Hidalgo, México. Sist Biocognitivos Tradic Paradig en la Conserv Biológica y el Fortalec Cult.

[37] Alonso-Aguilar LE, Montoya A, Kong A, Estrada-Torres A, Garibay-Orijel R (2014). The cultural significance of wild mushrooms in San Mateo Huexoyucan, Tlaxcala, Mexico. J Ethnobiol Ethnomed.

[38] Robles-García D, Suzán-Azpiri H, Montoya-Esquivel A, García-Jiménez J, Esquivel-Naranjo EU, Yahia E (2018). Ethnomycological knowledge in three communities in Amealco, Quéretaro, México. J Ethnobiol Ethnomed.

[39] Peña-Cañon ER, Enao-Mejía LG (2014). Conocimiento y uso tradicional de hongos silvestres de las comunidades campesinas asociadas a bosques de roble (Quercus humboldtii) en la zona de influencia de la Laguna de Fúquene, Andes Nororientales. Etnobiologia..

[40] Ruan-Soto F (2018). Sociodemographic differences in the cultural significance of edible and toxic mushrooms among Tsotsil towns in the Highlands of Chiapas, Mexico. J Ethnobiol Ethnomed.

[41] Ammirati JF, Traquair JA, Horgen PA (1985). Poisonous Mushrooms of the Northern United States and Canada.

[42] Pacheco-Cobos L, Rosetti M, Hudson R (2009). A new method for tracking pathways of humans searching for wild, edible fungi. Micol Apl Int.

[43] Pacheco-Cobos L, Rosetti M, Cuatianquiz C, Hudson R (2010). Sex differences in mushroom gathering: Men expend more energy to obtain equivalent benefits. Evol Hum Behav.

[44] López-Domínguez J, Acosta-Pérez R, Fernández JA, López JC (2005). Descripción del Parque Nacional Malinche. Primera Ed. Biodivers. del Parq. Nac. Malinche.

[45] Montoya Adriana, Méndez-Espinoza Claudia, Flores-Rivera Rodrigo, Kong Alejandro E-TA. Hongos tóxicos de Tlaxcala libro.pdf. Nieto de Pascual Pola Cecilia, Camacho Morfín Francisco H-TT, editor. Instituto Nacional de Investigaciones Forestales Agrícolas y Pecuarias, Universidad Autónoma de Tlaxcala; 2007. https://scholar.google.es/scholar?hl=es&as_sdt=0,5&cluster=7918832703906215707

[46] INEGI. Censo General de Población y Vivienda Principales resultados por localidad. México; 2010. https://www.inegi.org.mx/programas/ccpv/2010/

[47] Montoya Esquivel A. Aprovechamiento de los hongos silvestres comestibles en el volcán de La Malinche. Tlaxcala. 2005;159 http://132.248.9.195/ptd2005/00387/0345191/Index.html.

[48] Romero-Contreras T (1998). Los temazcales de San Isidro Buen Suceso.

[49] Nava NR (2012). Las ideologías lingüísticas a favor del náhuatl en San Isidro Buensuceso, Tlaxcala.

[50] Cano-Contreras EJ, Medinaceli A, Sanabria OL, Argueta A. Código de Ética para la investigación, la investigación-acción y la colaboración etnocientífica en América Latina. Etnobiología. 2016;14(Suppl 1) https://cutt.ly/cgjXWwk.

[51] Rodríguez-Gómez G, Gil-Flores J, García-Jiménez E (1999). Metodología de la investigación cualitativa.

[52] Fine GA (2003). Towards a peopled ethnography: developing theory from group life. Ethnography..

[53] Cifuentes J, Villegas M, Pérez-Ramírez L, Lot A, Chiang F (1986). Hongos. Manual de Herbario: Administración y manejo de colección.

[54] Lodge DJ, Ammirati JF, O’Dell TE, Mueller GM (2004). Collecting and describing macrofungi. Biodivers fungi Invent Monit methods.

[55] Romagnesi H (1968). Les Russules d ´Europe et dÁfrique du Nord.

[56] Corner EJH. Supplement to a Monograph of Clavaria and Allied Genera. J Cramer Lehre. 1970. 10.2307/1218546.

[57] Petersen RH (1975). Ramaria subgenus Lentoramaria with emphasis on North American Taxa. Bibl Mycol.

[58] Moser M, Kibby G (1983). Keys to Agarics and Boleti: Polyporales, Boletales, Agaricales, Russulales.

[59] Estrada-Torres A (1994). La familia gomphaceae (Aphyllophorales, fungi) en el estado de Tlaxcala. Tesis de Doctorado, Escuela Nacional de Ciencias Biológicas, Instituto Politécnico Nacional.

[60] Tulloss RE (1994). Seminario sobre Amanita.

[61] Kong A (1995). Estudio taxonómico sobre el género Lactarius (Russulales, Mycetae) en el Volcan La Malintzi, Tlaxcala.

[62] Kong A (2003). El género Russula (Fungi, Russulales) en el Parque Nacional La Malinche.

[63] Woods P (1987). La escuela por dentro: la etnografia en la investigacion educativa.

[64] Sandoval C (2002). Investigación cualitativa. Programa de especialización en teoría, métodos y técnicas de investigación social.

[65] Malinowski B. Los argonautas del Pacífico Occidental: comercio y aventura entre los indígenas de la Nueva Guinea Melanésica. Ediciones Península. 2001; http://eva.fhuce.edu.uy/file.php/194/63654554-Los-Argonautas-Del-Pacifico-Occidental-Vol-1-Bronislaw-Malinowski.pdf.

[66] De Munck VC, Sobo EJ (1998). Using methods in the field: a practical introduction and casebook.

[67] Bernard HR (1988). Research methods in cultural Anthropology: qualitative and quantitative aproaches.

[68] Bernard RH, Ryan GW (1998). Qualitative and quantitative methods of text analysis.

[69] Alexiades MN, Sheldon JW (1996). Selected guidelines for ethnobotanical research: a field manual.

[70] Lincon YS, Guba EG (1985). Naturalistic Inquiry Sage Beverly Hills. CA.

[71] Ryan GW, Nolan JM, Yoder PS (2000). Successive free listing: using multiple free lists to generate explanatory models. Field Methods.

[72] Weller SC, Romney AK (1988). Systematic data collection (Volumen 10).

[73] Durkheim É (2012). Las formas elementales de la vida religiosa: el sistema totémico en Australia (y otros escritos sobre religión y conocimiento).

[74] Kotowski MA, Pietras M, Luczaj L (2019). Extreme levels of mycophilia documented in Mazovia, a region of Poland. J Ethnobiol Etnomed.

[75] Tibuhwa DD (2012). Folk taxonomy and use of mushrooms in communities around Ngorongoro and Serengeti National Park, Tanzania. J Ethnobiol Ethnomed.

[76] Graeme KA (2014). Mycetism: A Review of the Recent Literature. J Med Toxicol.

[77] Lincoff G, Mitchel DH (1977). Toxic and hallucinogenic mushroom poisoning. A handbook for physicians and mushroom hunters.

[78] Harris M (1982). El materialismo cultural.

[79] Estrada-Torres A, Aroche RM (1987). Acervo etnomicológico en tres localidades del municipio de Acambay, Estado de México. Rev Mex Micol.

[80] Moreno Fuentes Á, Aguirre Acosta E, Pérez Ramírez L (2004). Conocimiento tradicional y científico de los hongos en el estado de Chihuahua, México. Etnobiología.

[81] Guzmán G (1997). Los nombres de los hongos y lo relacionado con ellos en América Latina. introducción a la etnomicobiota y micología aplicada de la región, sinonimia vulgar y científica. Xalapa, Veracruz, México.

[82] Ruan-Soto F (2018). Recolección de hongos comestibles silvestres y estrategias para el reconocimiento de especies tóxicas entre los tsotsiles de Chamula, Chiapas, México. Sci Fungorum.

[83] Guzmán-H G (1959). Sinopsis de los conocimientos sobre los hongos alucinógenos mexicanos. Boletín Soc Botánica México.

[84] Wasson RG, Garrido F (1983). El hongo maravilloso: Teonanácatl; Micolatría en Mesoamérica. Fondo de Cultura Económica.

[85] Costa-Neto E, Santos-Fita D, Vargas-Clavijo M (2009). Manual de Etnozoología.

[86] Zent EL. “We Come From Trees”: The Poetics of Plants among the Jotï of the Venezuelan Guayana. J Study Relig Nat Cult. 2009;3(1). 10.1088/1748-9326/8/1/015008.

[87] Bautista-González JA (2013). Conocimiento tradicional de hongos medicinales en seis localidades diferentes del país.

[88] Michelot D, Melendez-Howell LM (2003). Amanita muscaria: Chemistry, biology, toxicology, and ethnomycology. Mycol Res.

[89] Turner NJ (1988). “The importance of a rose”: evaluating the cultural significance of plants in Thompson and Lillooet Interior Salish. Am Anthropol.

