# Supplementary material for: Breaking the paradigms of residual categories and neglectable importance of non-used resources: the “vital” traditional knowledge of non-edible mushrooms and their substantive cultural significance
Source: J Ethnobiol Ethnomed. 2021 Apr 21;17:28. doi: 10.1186/s13002-021-00450-3 (PMC8059252; doi:10.1186/s13002-021-00450-3)
Supplement: Supplementary file 1 — Additional file 1. List of taxa recognized as non-edible [file 13002_2021_450_MOESM1_ESM.docx]

| **Additional file 1.** List of taxa recognized as non-edible in two communities from Tlaxcala, Mexico | | | |
| --- | --- | --- | --- |
| Scientific name | Local name in Spanish, Nahuatl or both | Community^a^ | Edibility^b^ |
| *Agaricus* *sylvicola* (Vittad.) Peck  A. Montoya 2109 | *Pipilo* | FJM | Edible |
| *Agaricus* sp. 1  Ramírez-Terrazo 37 | *I-tlatla in āyoh-tzin* | SIBS | ? |
| *Calvatia* sp. 1  Ramírez-Terrazo 368 | *Chiteboro de veneno* | SIBS | ? |
| *Chlorophyllum* *molybdites* (G. Mey.) Massee  Ramírez-Terrazo 342 | *Estiércol-nanacatl*  *Totolita* | SIBS | Toxic |
| *Coprinus* sp. 1  Ramírez- Terrazo 253 | *Hongo de veneno, hongo venenoso* | SIBS | Toxic |
| *Cystoderma* aff. *amianthinum* (Scop.) Fayod  Ramírez-Terrazo 259 | *Cuerudo*  *Comalito* | FJM | Non-edible |
| *Lycoperdon perlatum* Pers.  A. Montoya 2096 | *Chiteburo de veneno rojito* | SIBS | Edible |
| *Lycoperdon* sp. 1  Ramírez-Terrazo 319 | *Panza de sabino venenoso* | FJM | ? |
| *A. augusta* Bojantchev & R. M. Davis  A. Montoya 2091, 2095, 2097  Ramírez- Terrazo 255, 264, 337 | *Amantecado amarillo*  *Amantecado venenoso*  *Amarillo*  *Cītlal-nanacatl café*  *Ojo de venado*  *Tōtol-te-nanacatl*  *Venado*  *Yema prieta* | FJM SIBS | Toxic |
| *Amanita* aff. *cinereoconia* G.F. Atk.  A. Montoya 2118  Ramírez-Terrazo 292 | *Ángel venenoso*  *Blanco venenoso*  *Cītlal-nanacatl blanco*  *Hongo blanco de veneno, hongo blanco venenoso* | FJM SIBS | Non-edible |
| *A. fulva* Fr.  A. Montoya 2012  Ramírez-Terrazo 293, 360 | *Cuah-tlamanil de veneno* | SIBS | Edible |
| *A. pantherina* (DC.) Krombh. A. Montoya 2123, 2124, 2125  Ramírez Terrazo 301, 323, 365 | *Cītlal-nanacatl blanco*  *Mantecado venenoso*  *Mantequilla venenosa/o*  *Venado* | FJM SIBS | Toxic |
| *A. rubescens* Pers.  Alonso-Aguilar 59  Montoya 2090, 2093, 2099, 2132  Ramírez- Terrazo 247 | *Cītlal-nanacatl*  *Cuah-tlamanil de veneno*  *I-tlatla in cītlal-nanacatl*  *Mantecada/o*  *Xo-pitzāhuac* | SIBS | Edible |

| **Additional file 1.** *Continued* | | | |
| --- | --- | --- | --- |
| Scientific name | Local name in Spanish, Nahuatl or both | Community^a^ | Edibility^b^ |
| *A. xylinivolva* Tulloss, Ovrebo & Halling  Montoya 2128, 2133  Ramírez-Terrazo 270, 271, 364, 371 | *Hongo blanco de veneno/venenoso*  *I-tlatla in huevo nanacatl*  *Xo-tomāhuatl* | FJM  SIBS | Non-edible |
| *Amanita* sp. 1  Ramírez-Terrazo 324 | *Mantecado venenoso* | FJM | ? |
| *Amanita* sp*.* 2  Ramírez-Terrazo 311 | *Yema blanca*  *Iztāc nanacatl de veneno* | FJM | ? |
| *Amanita* sp*.*  A. Montoya 2101 | *Cuah-tlamanil-tzin malo* | SIBS | ? |
| *Amanita* sp*.* 4  Alonso-Aguilar 75 | *Cītlal-nanacatl* | SIBS | ? |
| *Auriscalpium vulgare* Gray  Ramírez-Terrazo 269 | *Hongo blanco de veneno/so* | FJM | Non- edible |
| *Guepinia helvelloides* (DC.) Fr.  Ramírez-Terrazo 305 | *Hongo de veneno/so* | SIBS | Edible |
| *Phellodon niger* (Fr.) P. Karst  Ramírez-Terrazo 32 | *Hongo azul de palo venenoso* | FJM | Non-edible |
| *Sarcodon* sp. 1  Alonso-Aguilar 60, 68, 69  Ramírez-Terrazo 303, 304, 307, 310 | *I-tlatla in tlalpīltzal*  *Tlalpīltzal de veneno* | SIBS | ? |
| *Baorangia* aff. *bicolor* (Kuntze) G. Wu, Halling & Zhu L. Yang  A. Montoya 2129 | *I-tlatla in xo-tomāh*  *Xo-tomāh rabia* | SIBS | Edible |
| *Boletus* parasitado  Ramírez-Terrazo 285 | *Volcancito* | FJM | Non-edible |
| *Boletus* sp. 1  Ramírez-Terrazo 261 | *Panté cimarrón de veneno* | FJM | ? |
| *Neoboletus erythropus*  (Pers.) C. Hahn | *Hongo-rado*  *Xo-tomāh de veneno*  *Xo-tomāh-rabia* | FJM  SIBS | Edible |
| *Xerocomellus chrysenteron*  (Bull.) Šutara  Ramírez-Terrazo 278  Ramírez-Terrazo 288, 289 | *Xo-tomāh-rabia*  *Hongo-rado*  *Panté cimarrón de veneno* | FJM  SIBS | Edible |
| *Clavariadelphus truncatus* Donk  Ramírez-Terrazo 377 | *Bate* | SIBS | Edible |
| *Clavulina* sp. 1  Ramírez-Terrazo 320 | *Escobeta venenosa* | FJM | ? |
| *Clavulina* sp. 2  Ramírez-Terrazo 321 | *Escobeta venenosa* | FJM | ? |

| **Additional file 1.** *Continued* | | | |
| --- | --- | --- | --- |
| Scientific name | Local name in Spanish, Nahuatl or both | Community^a^ | Edibility^b^ |
| *Cortinarius* sp. 1  Ramírez-Terrazo 258, 336 | *Amargoso venenoso* | FJM | ? |
| *Cortinarius* sp. 2  Ramírez-Terrazo 326 | *Panté venenoso* | FJM | ? |
| *Cortinarius* sp. 3  Ramírez -Terrazo 244 | *Xōlētl de veneno* | SIBS | ? |
| *Cortinarius sp. 4*  A. Montoya 2016, 2137  Ramírez-Terrazo 256 | *Cācāx-nanacatl de veneno*  *Galleta-nanacatl* | *SIBS* | *?* |
| *Cortinarius* sp. 5  A. Montoya 2136 | *Galleta-nanacatl* | SIBS | ? |
| *Cortinarius* sp. 6  Alonso-Aguilar 62 | *Hongo morado de veneno* | SIBS | ? |
| *Cortinarius* sp. 7  A. Montoya 2103 | *Estiercol-nanacatl* | SIBS | ? |
| *Cortinarius sp. 8*  A. Montoya 2107 | *Xocoyolin de veneno* | SIBS | ? |
| *Hemistropharia albocrenulata* (Peck) Jacobsson & E. Larss.  Ramírez-Terrazo 309 | *Hongo de veneno/so* | SIBS | Non-edible |
| *Entoloma* sp. 1  A. Montoya 2138 | *I-tlatla in xōlētl* | SIBS | ? |
| *Geastrum saccatum* Fr*.*  A. Montoya 2135 | *I-tlatla in chiteboro* | SIBS | Non-edible |
| *Ramaria abietina* (Pers.) Quél.  A. Montoya 2143 | *I-tlatla in xelhuāz* | SIBS | Non-edible |
| *R. gracilis* (Pers.) Quél.  Ramírez-Terrazo 358, 373 | *Una de ratón, Xelhuāz de veneno* | SIBS | Edible |
| *Helvella macropus* (Pers.) P. Karst.  A. Montoya 2139 | *Cuā-te-cax-nanacatl* | SIBS | Non-edible |
| *Helvella* sp. 1  Ramírez-Terrazo 361 | *Cola* | SIBS | ? |
| *Laccaria trichodermophora* G.M. Muell.  Ramírez-Terrazo 318  Ramírez-Terrazo 367 b | *Xocoyulado venenoso*  *Hongo de veneno/so* | FJM  SIBS | Edible |
| *Hygrocybe* sp. 1  Ramírez-Terrazo 262 | *Clavitos* | FJM | ? |
| *Hygrophorus purpurascens*  (Alb. & Schwein.) Fr  A. Montoya 2120, 2122 | *Camarón* | FJM | Edible |
| *Hygrophorus russula* (Schaeff.) Kauffman  Ramírez-Terrazo 314 | *Hongo venenoso* | FJM | Edible |

| **Additional file 1.** *Continued* | | | |
| --- | --- | --- | --- |
| Scientific name | Local name in Spanish, Nahuatl or both | Community^a^ | Edibility^b^ |
| *Hygrophorus* sp. 1  S/N | *Iztāc nanacatl de veneno* | SIBS | ? |
| *Hygrophoropsis aurantiaca*  (Wulfen) Maire  Ramírez-Terrazo 246, 274, 275, 27, 300  A. Montoya 2075, 2077, 2141 | *Camarón*  *Brindis*  *I-tlatla in te-cōzah*  *Te-cōzah cimarrón*  *Te-cōzah de veneno*  *Te-cōzah de veneno/sa*  *Tlapal-te-cōzah de veneno* | FJM  SIBS | Edible |
| *Coltricia* sp. 1  Ramírez-Terrazo 277 | *Ocō-xālito de veneno/so* | FJM | Non-edible |
| *Trametes* sp. 1  Ramírez-Terrazo 266 | *Cucharita*  *Hongo de palo de veneno* | FJM | Non-edible |
| *Inocybe* sp. 1  Ramírez-Terrazo 316 | *Hongo venenoso* | FJM | ? |
| *Inocybe* sp. 2  Ramírez-Terrazo 317 | *Hongo venenoso* | FJM | ? |
| *Inocybe* sp. 3  Ramírez-Terrazo 333 | *Xōlētl de burro* | FJM | ? |
| *Lyophyllum* gpo. *decastes*  Ramírez-Terrazo 341 | *Xōlētl de veneno* | SIBS | Edible |
| *Lyophyllum* sp. 1  Ramírez-Terrazo 332 | *Hongo venenoso* | FJM | ? |
| *Lyophyllum* sp. 2  Ramírez-Terrazo 283  A. Montoya 2122a | *Clavo*  *Hongo blanco de veneno/venenoso*  *Ruleta* | FJM | ? |
| *Lyophyllum* sp. 3  A. Montoya 2126b | *Hongo venenoso* | FJM | ? |
| *Gymnopus dryophilus* (Bull.) Murrill  Ramírez- Terrazo 251, 366 | *Sombrerito*  *Xōlētl de veneno* | SIBS | Edible |
| *Rhodocollybia butyracea* (Bull.) Lennox.  Ramírez-Terrazo 367 | *Hongo de veneno/so* | SIBS | Edible |
| *R. maculata* (Alb. & Schwein.) Singer  Alonso-Aguilar 74  A. Montoya 2076, 2079, 2114, 2117 | *Camarón*  *Hongo de veneno/so*  *Naranja*  *Paloma*  *Señorita venenosa* | FJM  SIBS | Non-edible |
| *Hohenbuehelia* sp. 1  Ramírez- Terrazo 249 | *Hongo de veneno/so* | SIBS | ? |
| *Psathyrella candolleana* (Fr.) Maire  Ramírez-Terrazo 280 | *Roto* | FJM | Edible |

| **Additional file 1.** *Continued* | | | |
| --- | --- | --- | --- |
| Scientific name | Local name in Spanish, Nahuatl or both | Community^a^ | Edibility^b^ |
| *Psathyrella* sp. 1  Ramírez Terrazo 331 | *Clavo de oyamel*  *Xōlētl de veneno* | FJM | ? |
| *Lactarius luculentus* Burl  Ramírez-Terrazo 302 | *Chīl-nanacatl de veneno* | SIBS | Non-edible |
| *L. mexicanus* A. Kong & Estrada  A. Montoya 2140  Ramírez-Terrazo 290 | *Cuā-te-cax de veneno I-tlatla in cuā-te-cax* | SIBS | Non-edible |
| *L. smithii* Montoya & Bandala  A. Montoya 2108, 2116 | *Camarón*  *Corneta venenosa* | FJM | Non-edible |
| *L. vinaceorufescens* A.H. Sm  Alonso-Aguilar 63, 64  A. Montoya 2080 | *Chīl-nanacatl de veneno-*  *I-tlatla in chīl-nanacatl*  *Enchilado cimarrón* | FJM  SIBS | Toxic |
| *Russula densifolia* Secr. ex Gillet  A. Montoya 2119 | *Corneta venenosa* | FJM | Non-edible |
| *R. emetica* (Schaeff.) Pers.  Ramírez-Terrazo 295 | *Hongo de veneno/so* | SIBS | Toxic |
| *R. hydrophila* Horniček  Alonso-Aguilar 65 | *Brindis* | SIBS | Non-edible |
| *R. romagnesiana* Shaffer  Ramírez-Terrazo 282 | *Corneta blanca malo* | FJM | Edible |
| *R. sancti-pauli* Kong & Buyck  Alonso-Aguilar 72  A. Montoya 2073, 2127 | *Brindis moradito*  *Camarón, Moradito, Paloma*  *Señorita venenosa*  *Tlilnanácatl* | FJM  SIBS | Edible |
| *R. viscida* Kudřna  A. Montoya 2094 | *Brindis* | SIBS | Non-edible |
| *Russula* sp. 1  A. Montoya 2100 | *Cuā-te-cax de veneno* | SIBS | ? |
| *Russula* sp. 2  A. Montoya 2014 | *Hongo de veneno/so* | SIBS | ? |
| *Agrocybe* sp. 1  Ramírez-Terrazo 284 | *Hongo de palo de veneno*  *Ocō-xālito blanco* | FJM | ? |
| *Hypholoma fasciculare* Massee  Ramírez-Terrazo 250, 286, 334  A. Montoya 2134 | *Amarillo*  *I-tlatla in tetecuin*  *Ojo de venado*  *Pata de gallina venenosa* | FJM  SIBS | Toxic |
| *Hypholoma* sp. 1  Ramírez-Terrazo 329 | *Hongo de mata venenoso* | FJM | ? |
| *Pholiota highlandensis* (Peck) Singer  Ramírez-Terrazo 267 | *Ocō-xālito café*  *Ocō-xālito de veneno/venenoso* | FJM | Toxic |
| *P. lenta* (Pers.) Singer  Ramírez-Terrazo 313 | *Patita de pájaro* | FJM | Edible |

| **Additional file 1.** *Continued* | | | |
| --- | --- | --- | --- |
| Scientific name | Local name in Spanish, Nahuatl or both | Community^a^ | Edibility^b^ |
| *P. lenta* (Pers.) Singer  Ramírez-Terrazo 313 | *Patita de pájaro* | FJM | Edible |
| *Pholiota* sp. 1  Ramírez-Terrazo 325 | *Xōlētl de veneno* | FJM | ? |
| *Pholiota* sp. 2  Ramírez-Terrazo 330 | *Hongo venenoso* | FJM | ? |
| *Suillus pseudobrevipes* A.H. Sm. & Thiers  Ramírez-Terrazo 268 | *Popozoh de veneno* | FJM | Edible |
| *S. tomentosus* (Kauffman) Singer  Ramírez-Terrazo 273 | *Pancita venenosa*  *Panza cimarrón* | FJM | Edible |
| *Clitocybe aff. crispa* H.E.  Bigelow & A.H. Sm.  Ramírez-Terrazo 322 | *Trompeta venenosa* | FJM | Non-edible |
| *Infundibulicybe gibba* (Pers.) Harmaja  A. Montoya 2142 | *I-tlatla in esquilon* | SIBS | Edible |
| *C. odora (*Bull.) P. Kumm.  Ramírez-Terrazo 347 | *Esquilon-nā-nanacatl* | SIBS | Edible |
| *Clitocybe* sp. 1  Ramírez-Terrazo 338 | *Ocō-xālito de veneno/so* | FJM | ? |
| *Clitocybe* sp. 2  Ramírez-Terrazo 312 | *Hongo de veneno/so* | FJM | ? |
| *Lepista nuda* (Bull.) Cooke  Ramírez-Terrazo 357a | *Hongo de veneno/so* | SIBS | Edible |
| *Leucopaxillus* sp. 1  Ramírez-Terrazo 306 | *Xōlētl de veneno* | SIBS | ? |
| *Tricholoma virgatum* (Fr.) P. Kumm  Ramírez-Terrazo 315, 374 | *Amargo*  *I-tlatla in caylita* | FJM  SIBS | Non-edible |
| *Tricholoma* sp. 1  Alonso- Aguilar 71 | *Iztāc nanacatl de veneno* | SIBS | ? |
| *Tricholoma* sp. 2  Alonso-Aguilar 61 | *Hongo de veneno/so* | SIBS | ? |
| ^a^ FJM Francisco Javier Mina, SIB San Isidro Buensuceso  ^b^ Based on information from the literaure  ? Edibility unknown | | | |
