# Supplementary material for: Breaking the paradigms of residual categories and neglectable importance of non-used resources: the “vital” traditional knowledge of non-edible mushrooms and their substantive cultural significance
Source: J Ethnobiol Ethnomed. 2021 Apr 21;17:28. doi: 10.1186/s13002-021-00450-3 (PMC8059252; doi:10.1186/s13002-021-00450-3)
Supplement: Supplementary file 2 — Additional file 2. Cultural importance of non-edible mushrooms in Francisco Javier Mina [file 13002_2021_450_MOESM2_ESM.docx]

| **Additional file** **2.** Cultural importance of non-edible mushrooms in Francisco Javier Mina, Tlaxcala, Mexico | | | | | |
| --- | --- | --- | --- | --- | --- |
| Scientific name | Traditional name  Ethnotaxon | Mention frequency | | Mention order | |
|  |  | M.N. | % M. | NV.MO | ROV |
| *Amanita muscaria* | **ajonjolinado** | **25** | **80.64%** | 19 (1^st^)  3 (2^nd^)  3 (4^th^) | **21.25** |
| *Pholiota* sp. 1  *Psathyrella* sp. 1 | **xolete de veneno** | **9** | **29.03%** | 3 (2^nd^)  2 (3^rd^)  2 (4^th^)  2 (5^th^) | **3.06** |
| *Neoboletus erythropus* | **hongo-rado** | **7** | **22.58%** | 2 (1^st^)  2 (2^nd^)  2 (3^rd^)  1 (4^th^) | **3.92** |
| *Tricholoma equestre* | **amargoso** | 6 | 19.35% | 1 (1^st^)  1 (2^nd^)  2 (4^th^)  1 (6^th^)  1 (7^th^) | 2.31 |
| *Suillus pseudobrevipes*  *S. tomentosus* | **popozoh venenoso** | 6 | 19.35% | 1 (2^nd^)  3 (3^rd^)  1 (4^th^)  1 (5^th^) | 1.95 |
| *Boletus* sp.1  *Xerocomellus chrysenteron* | **panté venenoso** | 5 | 16.13% | 3 (2^nd^)  2 (4^th^) | **2** |
| *Clavulina* sp. 1 y sp 2 | **escobeta de veneno** | 5 | 16.13% | 1 (1^st^)  1 (3^rd^)  1 (7^th^)  1 (8^th^)  1 (9^th^) | 1.71 |
| *Gyromitra ínfula* | **orejas de padre** | 5 | 16.13% | 1 (1^st^)  1 (4^rd^)  1 (8^th^)  1 (9^th^) | 1.49 |
| *Russula sancti-pauli* | **señoritas de veneno** | 5 | 16.13% | 1 (3^rd^)  2 (4^th^)  1 (6^th^)  1 (7^th^) | 1.14 |
| *A.* aff. *cinereoconia*  *A. xylinivolva*  *Lyophyllum* sp. 2 | **hongo blanco venenoso** | 5 | 16.13% | 1 (4^th^)  1 (5^th^)  1 (6^th^)  1 (7^th^)  1 (10^th^) | 0.86 |
| *Lactarius vinaceorufescens* | **enchilado malo** | 4 | 12.90% | 1 (1^st^)  1 (4^th^)  1 (6^th^)  1 (7^th^) | 1.56 |
| *Lactarius mexicanus* | **corneta de veneno** | 4 | 12.90% | 1 (2^nd^)  1 (3^rd^)  1 (4^th^)  1 (6^th^) | 1.25 |
| *Amanita pantherina* | **venadito venenoso** | 3 | 9.68% | 2 (2^nd^)  1 (7^th^) | 1.14 |
| *Hygrocybe* sp. 1 | **clavito malo** | 3 | 9.68% | 3 (5^th^) | 0.6 |
| MN: Mentions Number, *%* M*:* Mention proportion, NV MO*.:* Number of times mentioned in each order of mention, ROV*:* Ordinal value of rank | | | | | |
