# Supplementary material for: Breaking the paradigms of residual categories and neglectable importance of non-used resources: the “vital” traditional knowledge of non-edible mushrooms and their substantive cultural significance
Source: J Ethnobiol Ethnomed. 2021 Apr 21;17:28. doi: 10.1186/s13002-021-00450-3 (PMC8059252; doi:10.1186/s13002-021-00450-3)
Supplement: Supplementary file 3 — Additional file 3. Cultural importance of non-edible mushrooms in San Isidro Buensuceso [file 13002_2021_450_MOESM3_ESM.docx]

| **Additional file 3.** Cultural importance of non-edible mushrooms in San Isidro Buensuceso, Tlaxcala, Mexico | | | | | | |
| --- | --- | --- | --- | --- | --- | --- |
| Scientific name | Traditional name  Ethnotaxon | Mention frequency | | Mention order | |  |
|  |  | MN | % M | NV OM | OVR |  |
| *Boletus* aff. *bicolor*  *Neoboletus erythropus Xerocomellus chrysenteron* | **xo-tomāh-rabia** | 47 | 78.33% | 16 (1^st^)  12 (2^nd^)  7 (3^rd^)  6 (4^th^)  4 (5^th^)  1 (6^th^)  1 (15^th^) | 26.82 |  |
| *Amanita muscaria* | **cītlal-nanacatl** | 40 | 66.67% | 25 (1^st^)  8 (2^nd^)  3 (3^rd^)  1 (4^th^)  2 (5^th^)  1 (7^th^) | 30.79 |  |
| *Sarcodon* sp. 1 | **tlalpīltzal de veneno** | 27 | 45% | 3 (1^st^)  6 (2^nd^)  5 (3^rd^)  2 (4^th^)  3 (5^th^)  3 (6^th^)  2 (7^th^)  2 (9^th^)  1 (11^th^) | 9.87 |  |
| *Suillus* spp. | **popozoh de veneno** | 18 | 30% | 3 (1^st^)  5 (2^nd^)  4 (3^rd^)  1 (5^th^)  2 (6^th^)  1 (7^th^)  2 (8^th^) | 7.72 |  |
| *Ramaria abietina*  *R. gracilis* | **xelhuāz nanacatl de veneno** | 18 | 30% | 2 (3^rd^)  6 (4^th^)  5 (5^th^)  2 (6^th^)  1 (7^th^)  1 (12^th^)  1 (14^th^) | 3.79 |  |
| *Lactarius mexicanus* | **cuā-te-caxnanacatl de veneno** | 13 | 21.67% | 2 (2^nd^)  2 (3^rd^)  5 (4^th^)  2 (7^th^)  1 (10^th^)  1 (12^th^) | 3.39 |  |

| **Additional file 3**. *Continued.* | | | | | |
| --- | --- | --- | --- | --- | --- |
| Scientific name | Ethnotaxon | Frequency of mention | | Order of mention | |
|  |  | NM | % M | NV OM | OVR |
| *Hygrophoropsis aurantiaca* | **te-cōzah de pitzō-nanacatl** | 11 | 18.33% | 1 (1^st^)  2 (2^nd^)  3 (3^rd^)  1 (4^th^)  1 (6^th^)  2 (8^th^)  1 (9^th^) | 3.78 |
| *Agaricus* sp. 1  *Chlorophyllum molybdites* | **āyoh-tzin de veneno / estiercolnanacatl** | 7 | 11.66% | 1 (1^st^)  1 (4^th^)  1 (5^th^)  1 (7^th^)  2 (11^th^)  1 (16^th^) | 1.83 |
| *Clitocybe odora* | **esquilon-nā-nanacatl de veneno** | 6 | 10% | 1 (1^st^)  1 (2^nd^)  1 (4^th^)  1 (5^th^)  1 (6^th^)  1 (8^th^) | 2.24 |
| *Lactarius vinaceorufescens* | **chīl-nanacatl de pitzō-nanacatl /** **chīl-nanacatl de veneno** | 6 | 10% | 1 (3^rd^)  2 (4^th^)  1 (7^th^)  1 (13^th^)  1 (20^th^) | 1.1 |
| MN: Mentions Number, *%* M*:* Mention proportion, NV MO*:* Number of times mentioned in each order of mention, ROV*:* Ordinal value of rank | | | | | |
